# Metabolomic and Transcriptomic Changes in Porcine Cumulus–Oocyte Complexes Following Vitrification: Cysteinyl-Glycine as an Antioxidant Enhances the Quality of Vitrified Oocytes

**DOI:** 10.3390/antiox15101294

**Published:** 2026-10-05

**Authors:** Zhen He, Decai Xiang, Jing Fu, Demeng Mu, Xuemei Li, Junyu Jiang, Hongjiang Wei, Baoyu Jia, Guoquan Wu

**Affiliations:** 1 National Regional Genebank (Yunnan) of Livestock and Poultry Genetic Resources, Yunnan Provincial Engineering Laboratory of Animal Genetic Resource Conservation and Germplasm Enhancement, Yunnan Animal Science and Veterinary Institute, Kunming 650224, China; hezhen2026@163.com (Z.H.); askalm@163.com (D.X.);; 2 Key Laboratory for Porcine Gene Editing and Xenotransplantation in Yunnan Province, College of Veterinary Medicine, Yunnan Agricultural University, Kunming 650201, China

**Keywords:** vitrification, oocytes, cumulus cells, metabolomics, cysteinyl-glycine

## Abstract

Vitrification is an efficient approach to preserve porcine germinal vesicle (GV)-stage oocytes, yet cryo-induced damage remains a major limitation to subsequent embryonic development. Although previous studies have investigated transcriptomic and proteomic changes in oocytes and cumulus cells (CCs) following vitrification, the metabolic alterations underlying cryoinjury are poorly understood. In this study, metabolomic profiling of vitrified oocytes and CCs following in vitro maturation (IVM) was carried out identify key differential metabolites. Functional validation was further conducted by supplementing candidate metabolites during oocyte maturation. In vitrified oocytes, 19 metabolites were significantly upregulated, and 44 were significantly downregulated. Integrated metabolomic and transcriptomic profiling demonstrated that significantly altered metabolites and genes converged on biological pathways governing energy metabolism, glutathione metabolism, and glycerophospholipid metabolism. In CCs derived from vitrified oocytes, 70 metabolites exhibited marked upregulation, while 21 showed significantly downregulation. Integrated analysis profiling demonstrated that significantly altered metabolites and genes were converged on pathways such as glutathione metabolism and glycerophospholipid metabolism. Notably, oocytes and CCs shared 28 differentially expressed metabolites, among which cysteinyl-glycine (Cys-Gly) was significantly reduced in both oocytes and CCs following vitrification. Supplementing IVM with Cys-Gly improved vitrified oocyte quality, as evidenced by reduced oxidative stress, suppressed excessive apoptosis and aberrant autophagy, enhanced embryonic development, and increased cumulus expansion. Collectively, these findings provide a comprehensive metabolic profile of porcine GV oocytes and CCs after vitrification, offering new insights into the mechanisms of oocyte cryoinjury. Targeting key metabolic pathways or supplementing specific metabolites, such as Cys-Gly, may represent a promising strategy to improve the viability of vitrified porcine oocytes.

## 1. Introduction

Oocyte cryopreservation represents an essential strategy for preserving female genetic material in vitro [1]. Currently, vitrification is a common oocyte cryopreservation method [2]. Although continuous advances in vitrification technology have improved the cryosurvival rate of oocytes, the developmental potential of vitrified oocytes is still suboptimal. In pigs, vitrification of porcine germinal vesicle (GV) and metaphase-II (MII) oocytes has achieved relatively high survival rates [3,4]. However, vitrification at the MII stage of oocytes may disrupt the meiotic spindle and induce premature parthenogenetic activation, which compromises fertilization competence and blocks subsequent embryonic progression to the blastocyst [5,6]. In contrast, porcine oocytes vitrified at the GV stage retain the capacity for in vitro maturation (IVM) and fertilization and can develop into high-quality blastocysts and even full-term offspring [3]. Nevertheless, GV-stage vitrification still compromises developmental competence, indicating that vitrification causes physiological and molecular alterations. Omics technologies, including transcriptomics and proteomics, have facilitated the investigation of oocyte cryoinjury at the molecular level. Reproduction is intrinsically tied to the metabolic status of the organism. Tightly regulated energy metabolism is critical for proper oocyte development [7]. However, the metabolic consequences of vitrification in porcine oocytes are poorly understood.

Cumulus cells (CCs) are essential for oocyte development and maturation. As specialized granulosa cells, CCs communicate with oocytes through transzonal projections and gap junctions, thereby forming metabolically interconnected cumulus–oocyte complexes (COCs) [8,9]. Oocyte and CC interactions are influenced by multiple mechanisms, including transcriptional and metabolic signaling pathways [10]. In addition to supporting oocyte maturation, CCs help preserve the structural of oocytes during the vitrification process. As the first barrier to cryoprotective agents (CPAs), CCs help buffer osmotic stress and facilitate oocyte dehydration, thereby reducing oocyte cryodamage [11,12]. Despite these protective functions, exposure of COCs to CPAs or vitrification considerably reduces CC viability and induces DNA damage, compromising fertilization and embryo development [13]. Vitrification also disrupts communication between oocytes and CCs [14].

Metabolomics focuses on small molecule metabolites, the downstream products arising from gene expression and cellular metabolic processes [15]. Recently, metabolomics has been increasingly applied to investigate metabolic changes during oocyte maturation [16,17]. For example, untargeted metabolomic analyses have been used to characterize the metabolic profiles of oocytes and CCs across the follicular maturation stage within dominant follicles collected from young and aged mares [18]. Differential metabolomic signatures have also been identified between mural granulosa and cumulus cells in human preovulatory follicles [19]. Importantly, metabolomics may be applied independently or in combination with other omics approaches, including transcriptomics and proteomics, enabling the integration of multilayered information to construct gene regulatory networks and elucidate molecular interactions and causal relationships. Previous studies have combined proteomics and metabolomics to elucidate the mechanisms underlying mouse oocyte maturation, while others have integrated metabolomics and transcriptomics to characterize metabolic patterns during porcine oocyte development [20,21]. Changes in the metabolome in oocytes and CCs after vitrification can provide important clues for understanding the mechanisms of cryoinjury and finding protective strategies.

To date, the metabolic changes occurring in vitrified porcine GV-stage oocytes and their surrounding CCs post-IVM remain unknown. In the present work, we explored the transcriptomic and metabolic alterations in oocytes and CCs obtained from vitrified COCs following IVM using integrated untargeted metabolomic and transcriptomic analyses. Furthermore, we evaluated the effects of supplementing cysteinyl-glycine (Cys-Gly), a key differential metabolite, during IVM to determine its effects on the vitrified oocyte quality.

## 2. Materials and Methods

For this investigation, all chemicals and research reagents were sourced from Sigma-Aldrich Chemical Company (Shanghai, China), except where otherwise noted. The Tissue Culture Medium 199 (TCM199), Dulbecco’s phosphate-buffered saline (DPBS), CM-H2DCFDA, and ThiolTrackerTM Violet were acquired from ThermoFisher Scientific (Shanghai, China). Meanwhile, DAF-FM diacetate and the Lipid Peroxidation Assay Kit were secured through Beyotime (Shanghai, China).

### 2.1. COCs Collection

Ovaries were harvested from pubescent crossbred Landrace gilts at a commercial abattoir, then conveyed to our laboratory within 2 h in prewarmed saline (35–37 °C) supplemented with antibiotics-75 mg/L of penicillin G and 50 mg/L of streptomycin. Upon arrival, the ovaries specimens underwent a through double-rinse using the same saline containing antibiotics. COCs were aspirated from 3–8 mm antral follicles using an 18-gauge needle (Jiangxi Qiaoming Medical Device Co., Ltd., Yichun, China) mounted on a 20 mL disposable syringe (Jiangxi Qiaoming Medical Device Co., Ltd., Yichun, China). Harvested COCs underwent two washes in Tyrode’s lactate-HEPES-polyvinyl alcohol medium [22]. COCs were viewed using a stereomicroscope (Olympus, Tokyo, Japan), and oocytes with homogeneous cytoplasm and surrounding CCs were retained for subsequent experiments.

### 2.2. COCs Vitrification and Warming

The vitrification and thawing protocols were adopted from our previously published work [23]. All solutions were formulated with DPBS supplemented with 20% (*v*/*v*) synthetic serum substitute (Irvine Scientific, Santa Ana, CA, USA), defined as the basic medium (BM). Before vitrification, COCs were rinsed with BM and incubated in a 5% (*v*/*v*) ethylene glycol (EG) solution to achieve equilibration, lasting 10 min at 25 °C. Subsequently, batches containing roughly 10–15 COCs were moved into vitrification medium prepared in BM, which contained 0.6 M sucrose, 50 mg/mL polyvinylpyrrolidone, and 35% (*v*/*v*) EG, and exposed for 20–30 s at 25 °C. COCs were then mounted on the tip of a Cryotop carrier (Kitazato Biopharma, Shizuoka, Japan) and directly placed in liquid nitrogen (LN_2_). For warming, Cryotop-loaded COCs were retrieved from LN_2_ and immersed in 1.0 M sucrose solution for 1 min at 42 °C. The samples were then sequentially exposed to 0.5 and 0.25 M sucrose solutions, with 2.5 min incubation in each solution. Afterwards, COCs were flushed in BM for an additional 5 min before undergoing IVM culture.

### 2.3. Oocyte IVM

IVM was performed in a 24-well plate (Costar, Corning, NY, USA). Each well containing 500 µL of IVM solution overlaid with mineral oil for 42–44 h in a 5% CO_2_ incubator (Thermo Scientific, Waltham, MA, USA) at 39 °C [24]. IVM medium was prepared by supplementing TCM199 with 10% (*v*/*v*) porcine follicular fluid, 0.57 mM cysteine, 3.05 mM D-glucose, 0.91 mM sodium pyruvate, 10 ng/mL epidermal growth factor, and 0.01 units/mL each of follicle stimulating hormone and luteinizing hormone. Approximately 50–70 oocytes were cultured per well. After IVM, oocytes were carefully stripped from CCs by repeatedly pipetting them through a solution containing 0.1% (*w*/*v*) hyaluronidase. Oocyte quality was then assessed via morphological inspection under a microscope. Non-viable oocytes were distinguished based on disrupted oolemma alongside brownish-yellow cytoplasm, while viable oocytes exhibited a smooth, bright oolemma and evenly distributed dark cytoplasm. Nuclear maturation was defined by extrusion of the first polar body. Following hyaluronidase digestion, CCs were collected, centrifugated for 3 min at 1000 rpm, and rinsed three times with pre-chilled PBS. After removal of the supernatant, the CCs were snap-frozen in LN_2_.

### 2.4. Metabolite Extraction

After IVM, both matured oocytes at the MII stage and their corresponding CCs were collected, promptly cryopreserved in LN_2_, and maintained at −80 °C. For metabolite extraction, each frozen sample was thawed on ice and mixed with 120 µL precooled 50% methanol per 20 µL sample. After thorough vortexing for 1 min, the mixtures were incubated for 10 min and then kept at −20 °C overnight. Following centrifugation at 4000× *g*, the resulting supernatant was transferred to 96-well plates and stored at −80 °C until liquid chromatography–mass spectrometry (LC-MS) analysis. Quality control (QC) samples were generated by combining 10 μL aliquots from each extraction mixture.

### 2.5. Metabolomic Data Processing

The raw data were transformed into mzXML files and subsequently analyzed using XCMS, CAMERA, and the metaX R package. Metabolite features were annotated by matching against in-house and public databases. The Kyoto Encyclopedia of Genes and Genomes (KEGG) and the Human Metabolome Database (HMDB) were used to annotate the metabolites. Principal component analysis (PCA) was applied to the preprocessed dataset to detect outliers. Probabilistic quotient normalization was applied across all samples, followed by QC-based robust spline correction to remove bath variation. PLS-DA was implemented via metaX to detect metabolic differences between groups. Metabolites displaying *p* < 0.05, fold change (FC) > 1.2 or FC < 0.8, and VIP > 1were defined as significantly altered metabolites.

### 2.6. RNA-seq Analysis

RNA-seq was carried out following our published protocol [25]. Harvested MII oocytes and CCs were plunged into cell lysis buffer, snap-frozen immediately in LN_2_, and stored at −80 °C. Full-length cDNA was synthesized with the SMART-Seq v4 Ultra Low Input RNA Kit (Takara Bio, Mountain View, CA, USA). Libraries were sequenced on the Illumina NovaSeq™ 6000 platform (Illumina, San Diego, CA, USA) with 150 bp paired-end reads (LC-Bio Sciences, Hangzhou, China). Differentially expressed genes (DEGs) were pinpointed through Cuffdiff (version 2.2.1), which relies on a negative binomial distribution framework. A gene was deemed to exhibit significant differential expression if it met the thresholds of a *p*-value < 0.05 and FC > 1.5 or < 0.67. DEGs were then subjected to KEGG enrichment analysis. Functional enrichment analysis was performed using the DAVID Bioinformatics Resource by uploading the list of significant DEGs into the functional annotation module to identify enriched KEGG pathways.

### 2.7. Metabolomic and Integrated Metabolome–Transcriptome Analysis

Pathway enrichment analysis of differential metabolites was conducted using MetaboAnalyst (http://www.metaboanalyst.ca//upload/JointUploadView.xhtml, version 6.0, 25 September 2024) [26]. In the “Pathway Analysis” module, differential metabolites were uploaded for pathway enrichment and topology analyses. Metabolic pathway enrichment bubble plots were generated with log(*p*) values displayed on the *y*-axis and pathway impact values on the *x*-axis. Pathway impact scores were calculated based on topological analysis, with pathways exhibiting higher impact values and located at the upper-right corner of the bubble plot considered the most relevant.

Integrated metabolic pathway analysis of metabolomic and transcriptomic datasets was conducted using the “Joint Pathway Analysis” module.

### 2.8. Evaluation of the Cumulus Expansion Index (CEI)

CEI was determined by morphological assessment of COCs after 44 h of IVM, as previously described [27]. COCs were classified into five grades: grade 0, no expansion with detachment of CCs and exposure of the oocyte; grade 1, no expansion but CCs tightly surround the oocyte; grade 2, expansion limited to the outermost CC layer; grade 3, expansion in all CC layers except the corona radiata; grade 4, maximum expansion involving the corona radiata. The CEI was computed using the formula: CEI = [(0 × grade 0 COCs) + (1 × grade 1 COCs) + (2 × grade 2 COCs) + (3 × grade 3 COCs) + (4 × grade 4 COCs)]/total number of cultured COCs.

### 2.9. Oocyte Staining with Fluorescent Probes

To measure the levels of intracellular reactive oxygen species (ROS), lipid peroxidation (LPO), nitric oxide (NO), and glutathione (GSH), oocytes were incubated at 39 °C for 30 min in DPBS-PVA medium supplemented with 10 μM CM-H2DCFDA, 2 μM BDPY 581/591 C11, 5 μM DAF-FM diacetate, or 10 μM ThiolTrackerTM Violet. After incubation, oocytes were rinsed three times using DPBS-PVA and transferred into 100 μL of DPBS-PVA in glass-bottom culture dishes for imaging. Fluorescence signals were detected using a confocal laser-scanning microscope (Nikon A1, Nikon, Tokyo, Japan) with excitation wavelengths of 488 nm (green) for ROS and NO, 405 nm (blue) for GSH, and 535 nm (red) for LPO.

Oocytes were stained with FITC-DEVD-FMK (BioVision, Mountain View, CA, USA) and MR-RR2 (Immunochemistry, Bloomington, MN, USA) at working concentrations of 1:300 and 1:250, respectively, to assess the activities of activated caspase-3 and cathepsin B, which reflect the apoptotic potential; autophagy was assessed using Cyto-ID Green (Enzo Life Sciences, Farmingdale, NY, USA; 1:1000) and LysoTracker™ Red (50 nM), which specifically label autophagic compartments and lysosomes, respectively. The incubation and imaging procedures were identical to those described for the ROS assay. Fluorescence signals were detected using a confocal laser-scanning microscope with excitation wavelengths of 488 nm (green) for FITC-DEVD-FMK and Cyto-ID Green, and 535 nm (red) for MR-RR2 and LysoTracker Red.

Fluorescence probe staining was performed on 8–10 oocytes per group in three independent experiments. All oocytes with the same fluorescent probe were imaged under the same scanning conditions. Image analysis was completed with NIS-Elements software (4.50.00) (Nikon, Tokyo, Japan). The mean fluorescence intensity of individual oocyte was calculated as total fluorescent signal divided by oocyte area. The average fluorescence intensity of the vitrification group was set to 1, and the fluorescence values from the remaining groups were compared with this reference value.

### 2.10. qRT-PCR

Total cDNA was prepared from oocytes or CCs utilizing the TransScript UniCell to cDNA Synthesis SuperMix for qPCR (TransGen Biotech, Beijing, China) following the manufacturer’s instructions. For each sample, 25 MII oocytes, or the CCs collected from the corresponding 25 COCs, were used, four biological replicates per group. qRT-PCR was run on a CFX Real-Time PCR Detection System (Bio-Rad, Hercules, CA, USA) with Fast qPCR Mix (Tsingke, Beijing, China). Each reaction setup included four replicates. Target gene expression was quantified relative to the GAPDH reference gene using the 2^−ΔΔCT^ calculation method. Primer sequences utilized throughout this study are provided in Supplementary Table S1.

### 2.11. Parthenogenetic Activation (PA)

PA were performed according to standard procedures [28]. MII oocytes were exposed to the activation solution (0.28 M mannitol, 0.1 mM MgSO4, 0.05 mM CaCl2 and 0.5 mM HEPES) for 30 s and then transferred into a microslide fusion chamber prefilled with the activation solution. Electrical activation was performed using a BLS CF-150/B Cell Fusion Machine (Budapest, Hungary), in which oocytes underwent a direct-current pulse of 1.3 kV/cm for 80 μs. After electrical activation, oocytes were maintained in a porcine zygote medium-3 (PZM-3) supplemented with cytochalasin B and cycloheximide for 4 h. Subsequently, embryos were transferred to PZM-3. Embryo culture was performed at 39 °C within a humidified incubator with 5% CO_2_. On Day 5 post-PA, 10% fetal bovine serum was added to the PZM-3 medium. Cleavage and blastocyst formation rates were assessed on Days 2 and 6 post-PA, respectively (PA = Day 0).

### 2.12. Experimental Design

Experiment 1: Metabolome and transcriptome data analysis of oocytes and CCs. For this experiment, the COCs obtained from each batch were allocated into two experimental groups. Approximately 40% of the total COCs went straight into IVM, while the remainder underwent vitrification, warming, and subsequent IVM. After IVM, oocytes and their CCs from non-vitrified COCs were harvested separately as the control cohort (fresh oocytes (FOs), fresh CCs (FCs)); those originating from vitrified COCs formed the treatment group (vitrified oocytes (VOs), vitrified CCs (VCs)). Each metabolomic replicate contained approximately 800 MII oocytes, while each transcriptomic replicate contained approximately 10 MII oocytes. We separately analyzed differentially expressed metabolites and genes in fresh and vitrified oocytes, performing KEGG enrichment analysis on each group. Subsequently, we conducted joint analysis of metabolome and transcriptome data from fresh and vitrified oocytes. CC data were analyzed similarly to oocyte data.

Experiment 2: Validation of the developmental capacity of vitrified COCs supplemented with GSH or Cys-Gly. The supplementation concentrations were selected as follows: 4 mM GSH was chosen based on previous studies [29,30], whereas 2 mM Cys-Gly was determined based on the preliminary experimental results. Following vitrification and subsequent warming, COCs underwent maturation culture with or without GSH/Cys-Gly supplementation throughout the entire IVM process, corresponding to the Vitr, Vitr + GSH, and Vitr + Cys-Gly treatment groups. The study first assessed oocyte survival rate, nuclear maturation rate, and cumulus expansion degree under all experimental conditions. Following PA, the developmental potential of resulting MII oocytes was evaluated by monitoring cleavage rate and blastocyst formation rate. Subsequently, MII oocytes were assessed for oxidative stress, apoptosis, and autophagy by fluorescence probe staining and mRNA expression of related genes, whereas CCs were assessed for mRNA level related to oxidative stress and cumulus cell expansion.

### 2.13. Statistical Analysis

Data analysis was performed with GraphPad Prism (version 10). The results are expressed as least-squares mean ± standard error of the mean (SEM). Two-group comparisons were carried out via student’s *t*-test, whereas one-way ANOVA was adopted for comparisons across three experimental groups. A *p* < 0.05 was considered statistically significant (* *p* < 0.05, ** *p* < 0.01, *** *p* < 0.001, **** *p* < 0.0001). Graphs were also completed in GraphPad Prism (version 10). The graphical abstract was generated using BioGDP.com [31].

## 3. Results

Supplementary Table S1 data demonstrated vitrification reduced GV oocyte sur-vival and developmental potential. After warming, the survival rate of vitrified oocytes (85.6%) was markedly lower than that of fresh oocytes (100%), and it further declined to 80.5% after IVM (fresh: 89.3%). In contrast, the MII rates following IVM were comparable between the two groups (vitrified 85.5% vs fresh 88.8%). Following parthenogenetic activation, vitrified oocytes exhibited substantially lower cleavage (vitrified 73.2% vs fresh 88.1%) and blastocyst formation (vitrified 22.1% vs fresh 54.4%) rates than fresh oocytes.

### 3.1. Differential Metabolic Profiles of Vitrified and Fresh GV Oocytes and CCs post-IVM

**Figure 1:**
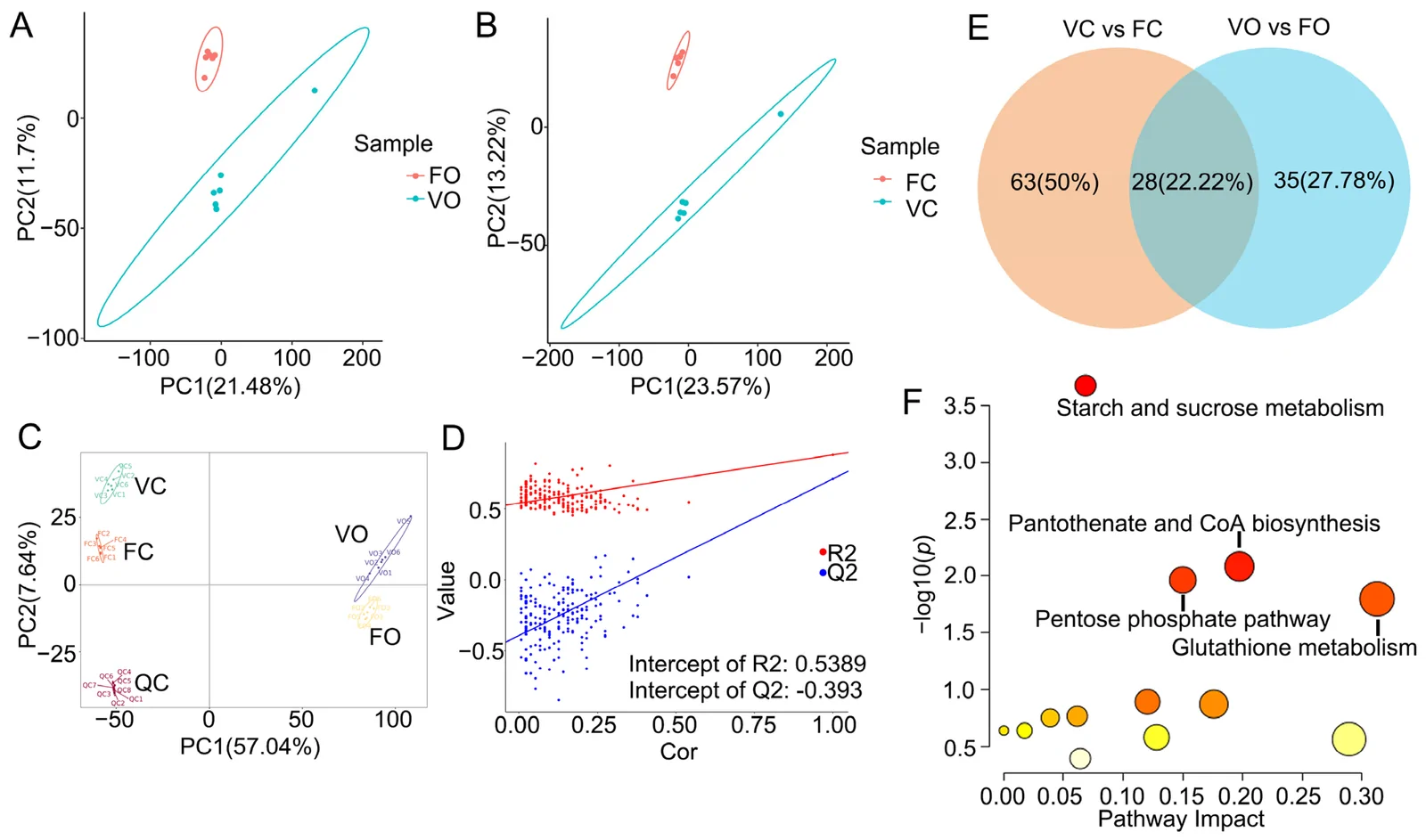
Differences in the metabolic characteristics of vitrified versus fresh porcine GV oocytes and cumulus cells post-IVM. (**A**,**B**) Principal component analysis of metabolic features. (**C**) Partial least squares discriminant analysis (PLS-DA). (**D**) Permutation test of the PLS-DA model. (**E**) Venn diagram indicating the number of VOs versus FOs and VCs versus FCs sharing differential metabolites. Differentially expressed metabolites were selected based on VIP > 1, *p* < 0.05, and FC < 0.8 or FC > 1.2. (**F**) Differential metabolite enrichment pathways shared by VO versus FO and VC versus FC. Circle color denotes pathway enrichment significance (−log10(p)), with size reflecting impact value.

Metabolic profiles of vitrified versus fresh porcine GV oocytes and CCs post-IVM were analyzed using UPLC-MS. A total of 478 metabolites were identified using untargeted metabolomics. Based on chemical classification in the HMDB database, 427 metabolites were further annotated across 13 categories. The predominant metabolite groups comprised lipids and lipid-like molecules, organic acids and derivatives, benzenoids, organoheterocyclic compounds, nucleosides, and organic oxygen compounds (Figure S1A). KEGG annotation indicated that these metabolites were involved in metabolic pathways, cellular processes, and disease-associated functions (Figure S1B). PCA revealed clear separation between vitrified and fresh groups, with clustering observed within biological replicates (Figure 1A,B). These results suggest differences in metabolic profiles between vitrified and fresh oocytes and surrounding CCs.

PLS-DA further identified significant differential metabolites (Figure 1C,D). A total of 63 differential metabolites were detected between vitrified and fresh GV oocytes after IVM (VO versus FO), while 91 differential metabolites were identified in CCs (VC versus FC) (Figure 1E). In addition, 28 differential metabolites were shared between oocytes and CCs. KEGG enrichment revealed that these shared metabolites primarily participate in glutathione metabolism, pantothenate and CoA biosynthesis, the pentose phosphate pathway, and starch and sucrose metabolism (Figure 1F and Figure S2). These results indicate strong intercellular communication and metabolic coupling between oocytes and surrounding CCs. The shared differential metabolites may be the key molecular mediators of this interaction.

### 3.2. Differential Metabolic Pathways of Vitrified and Fresh GV Oocytes

**Figure 2:**
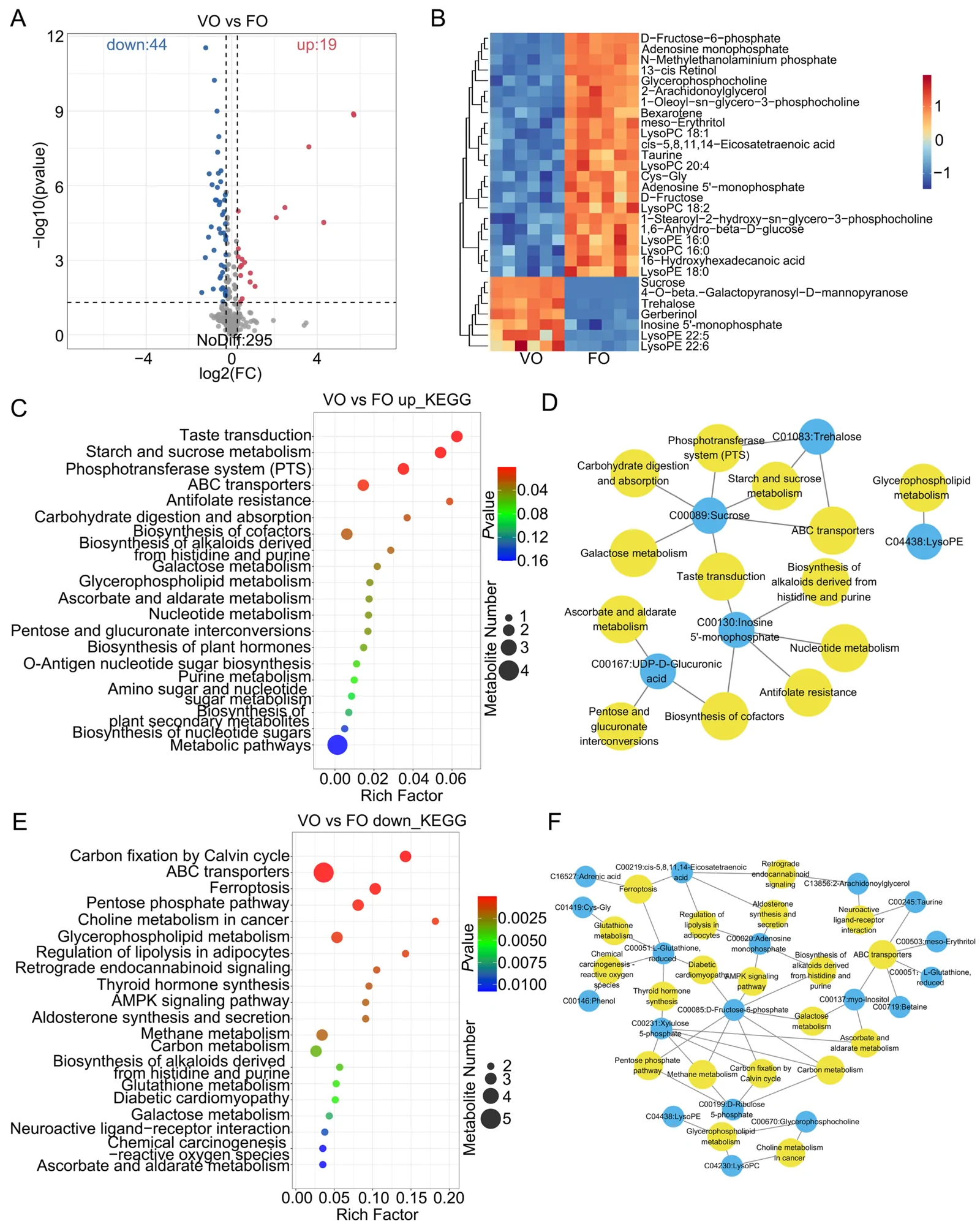
Differential metabolite profiling of vitrified versus fresh porcine GV oocytes post-IVM. (**A**) Volcano diagrams illustrating differential metabolites (VIP > 1, *p* < 0.05, and FC < 0.8 or FC > 1.2). The vertical dashed lines indicate the fold change threshold of 1.2, and the dashed horizontal line marks the cutoff for statistical significance at *p* = 0.05. (**B**) Heatmap of differential metabolites. (**C**) Bubble plot of the top 20 KEGG enrichment upregulated metabolites. Rich Factor indicates the number of differential metabolites located at that KEGG/total number of metabolites located at that KEGG. The smaller the *p*-value, the higher the KEGG enrichment. (**D**) Network map of upregulated metabolites and enriched pathways. Yellow nodes stand for metabolic pathways, and blue nodes denote differential metabolites. (**E**) Bubble plot of the top 20 KEGG enrichment downregulated metabolites. (**F**) Network map of downregulated metabolites and enriched pathways. Yellow nodes represent metabolic pathways, whereas blue nodes mark differential metabolites.

To further characterize metabolic alterations in oocytes, differential metabolites between vitrified and fresh GV oocytes were analyzed. 63 distinct metabolites emerged in VO versus FO, featuring 19 upregulated and 44 downregulated metabolites. Volcano plot and clustering heatmap analyses were adopted to visualize differential metabolite distribution and expression patterns across samples (Figure 2A,B). Significantly distinct expression patterns were observed between the groups, whereas samples within the same group exhibited similar expression profiles (Figure 2B). Based on metabolite structure and functional classification in the HMDB, the identified potential biomarkers were grouped into multiple families: lipids and lipid-like molecules, organic acids and derivatives, organic oxygen compounds, nucleosides, nucleotides, and their analogues, organooxygen compounds, benzenoids, phenylpropanoids, and polyketides (Figure S3A, Supplemental Table S2).

KEGG enrichment was subsequently carried out separately for upregulated and downregulated metabolites, and metabolite–pathway interaction networks were constructed (Figure 2C–F). In the VO versus FO comparison, pathways enriched by upregulated metabolites were mainly associated with carbohydrate metabolism and biosynthesis, including starch and sucrose metabolism, the phosphotransferase system, carbohydrate digestion and absorption, galactose metabolism, and pentose and glucuronate interconversions, as well as glycerophospholipid metabolism (Figure 2C,D, Supplemental Table S3). The metabolites that exhibited reduced expression were predominantly concentrated in biology pathways associated with ferroptosis, the pentose phosphate pathway, glycerophospholipid metabolism, AMPK signaling, and glutathione metabolism (Figure 2E,F, Supplemental Table S3).

### 3.3. Transcriptome Changes and Integrated Analysis of Differential Metabolites and Genes in Oocytes

**Figure 3:**
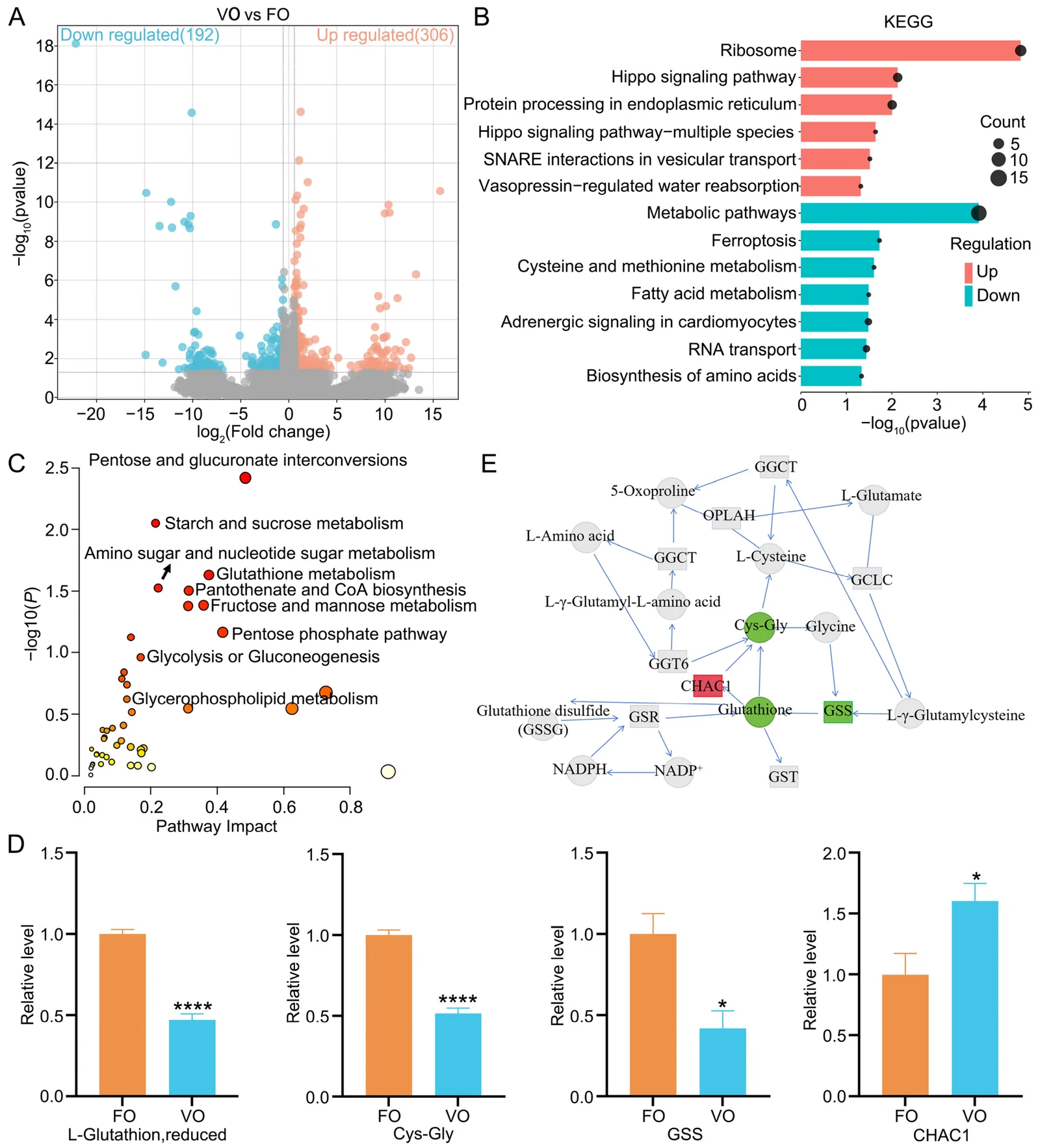
Transcriptomic profiling and integrated transcriptomic–metabolomic analysis of vitrified and fresh porcine GV oocytes post-IVM. (**A**) Volcano map depicting the total number of differentially expressed genes and the number of significantly upregulated and downregulated differentially expressed genes. The horizontal axis of the volcano map displays the change in gene expression, and the vertical axis indicates the level of gene significance. The orange dots highlight genes that have been upregulated, while the blue markers indicate those that have been downregulated, with the gray ones indicate genes with on significant change. DEGs were defined as *p* < 0.05 and FC < 0.67 or FC > 1.5. (**B**) Top enriched KEGG pathways for upregulated and downregulated DEGs. (**C**) Co-enriched KEGG pathways of differential metabolites and genes. (**D**) Relative levels of metabolites and genes involved in glutathione metabolism. Relative levels were calculated by dividing the value of each sample by the mean value of the FO group. Data are presented as the mean ± SEM. Panels for L-glutathione (reduced) and Cys-Gly represent metabolite relative abundance, whereas panels for GSS and CHAC1 represent relative mRNA expression. Student’s *t*-test served as the method for statistical evaluation. * *p* < 0.05, **** *p* < 0.0001. (**E**) Schematic diagram of glutathione metabolism. Circles represent metabolites, while rectangles denote genes. Green indicates downregulated metabolites or genes; red indicates upregulated metabolites or genes. Arrows indicate the biochemical reaction direction.

RNA-seq analysis was employed to evaluate transcriptional alterations in vitrified and fresh GV oocytes post-IVM. The comparison revealed a total of 498 DEGs, with 306 showed increased expression and 192 exhibiting decreased expression (Figure 3A). To delve deeper into the roles of these genes, we conducted a KEGG pathway analysis. Upregulated DEGs were particularly clustered into six KEGG pathways (Figure 3B, Supplemental Table S4), including the Hippo signaling pathway and protein processing in the endoplasmic reticulum. Downregulated DEGs were associated with seven KEGG pathways, such as metabolism pathways, ferroptosis, and fatty acid metabolism (Figure 3B, Supplemental Table S4).

To further examine vitrification’s effects on oocyte development, integrated transcriptomic and metabolomic analyses were performed. Integrated pathway analysis revealed seven significantly enriched pathways, primarily associated with carbohydrate metabolism and glutathione metabolism (Figure 3C). Further analysis of glutathione metabolism demonstrated that the metabolites Cys-Gly and GSH were downregulated in the vitrified oocytes. These alterations were accompanied by downregulation of glutathione synthase (GSS) and upregulation of ChaC glutathione-specific gamma-glutamylcyclotransferase 1 (CHAC1) (Figure 3D,E).

In addition, vitrification affected carbohydrate metabolism during oocyte maturation, including pathways related to starch and sucrose metabolism, glycolysis, the pentose phosphate pathway, amino sugar and nucleotide sugar metabolism, and pentose and glucuronate interconversions. Among the altered metabolites, D-fructose, D-fructose-6-phosphate, D-ribulose 5-phosphate, xylulose 5-phosphate, myo-inositol, N-acetyl-D-glucosamine 6-phosphate, and 3′-dephosphocoenzyme A were downregulated, whereas UDP-D-glucuronic acid and D-(+)-pantothenic acid were upregulated in vitrified oocytes (Figure S4A,B).

Porcine oocytes exhibited exceptionally high lipid content, and vitrification disrupted lipid metabolism, particularly glycerophospholipid metabolism. Among the lipid-associated metabolites, lysophosphatidylcholine (LysoPC) and lysophosphatidylethanolamine (LysoPE) were the most significantly affected and were downregulated following vitrification (Figure S5A,B).

### 3.4. Differential Metabolic Pathways in CCs Obtained from Vitrified and Fresh GV Oocytes

**Figure 4:**
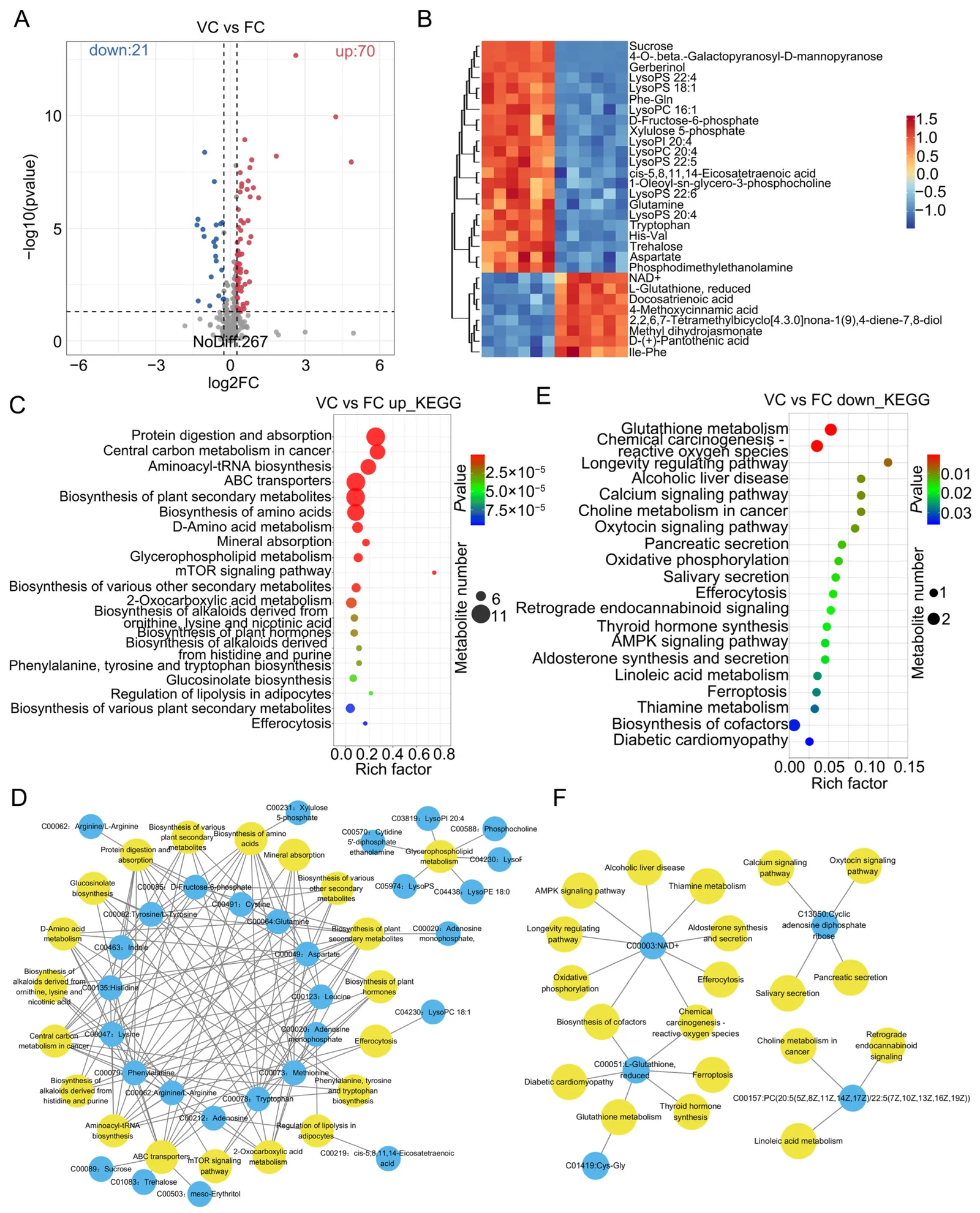
Differential metabolite profiling in CCs from vitrified versus fresh porcine GV oocytes post-IVM. (**A**) Volcano plots illustrating differentially abundant metabolites (VIP > 1, *p* < 0.05, and FC < 0.8 or FC > 1.2). (**B**) Heatmap showing the 30 metabolites with the most prominent expression differences. (**C**) Bubble plot of the top 20 KEGG enrichment upregulated metabolites. Rich Factor indicates the number of differential metabolites located at that KEGG/total number of metabolites located at that KEGG. The smaller the *p*-value, the higher the KEGG enrichment. (**D**) Network map of upregulated metabolites and pathways. Yellow nodes denote metabolic pathways, while blue nodes represent the differentially abundant metabolites. (**E**) Bubble plot of the top 20 KEGG enrichment downregulated metabolites. (**F**) Network map of downregulated metabolites and pathways. Yellow nodes denote metabolic pathways, while blue nodes represent the differentially abundant metabolites.

CCs are essential ovarian cells that play key roles in oocyte development and maturation. We identified 91 altered metabolites in CCs collected from vitrified versus fresh porcine GV oocytes after IVM: 70 upregulated and 21 downregulated metabolites (Figure 4A). Significant differences were observed in the expression signatures of candidate biomarkers between two groups, while samples within the same group exhibited similar expressions (Figure 4B). These potential biomarkers were categorized into several classes, including lipids and lipid-like molecules, organic oxygen compounds, nucleosides, nucleotides and their analogues, organoheterocyclic compounds, benzenoids, phenylpropanoids and polyketides, and organooxygen compounds (Figure S3A, Supplemental Table S5).

KEGG enrichment analysis was subsequently performed for upregulated and downregulated differential metabolites, and metabolite–pathway interaction networks were constructed (Figure 4C–F). Pathways enriched by the upregulated metabolites were mainly associated with protein digestion and absorption, amino acid metabolism, glycerophospholipid metabolism, and the mTOR signaling pathway (Figure 4C,D, Supplemental Table S6). The downregulated metabolites were mainly related to glutathione metabolism, the calcium signaling pathway, the oxytocin signaling pathway, oxidative phosphorylation, and the AMPK signaling pathway (Figure 4E,F, Supplemental Table S6).

### 3.5. Transcriptome Changes and Association Analysis of Metabolic Pathways for Differential Metabolites and Differential Genes in CCs

**Figure 5:**
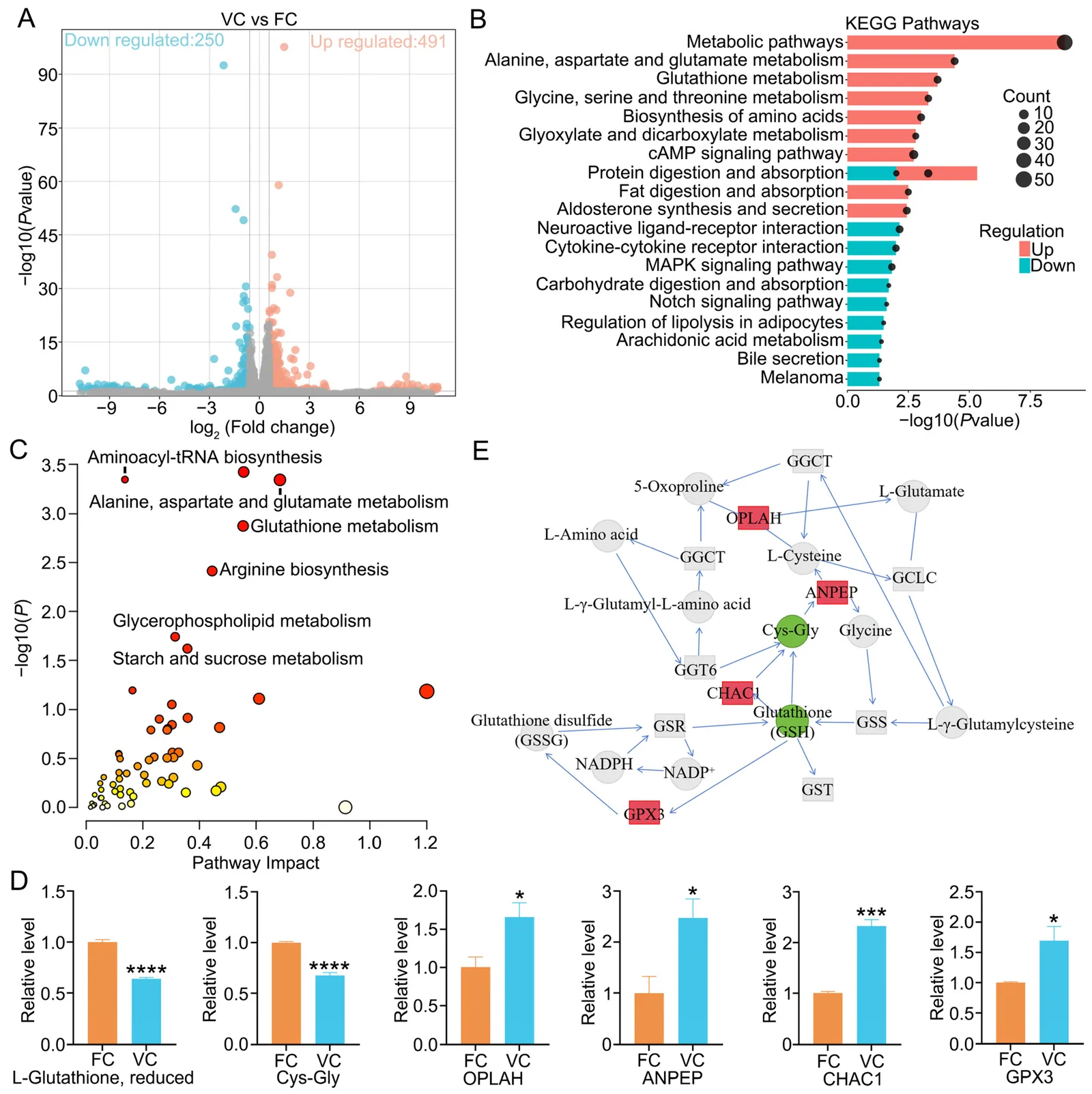
Integrated omics analysis of transcriptomes and metabolomes in CCs from vitrified versus fresh porcine GV oocytes following IVM. (**A**) Volcano map depicting the number of significantly upregulated and downregulated DEGs. The horizontal axis of the volcano map indicates the change in gene expression, and the vertical axis indicates the level of gene significance. The orange markers signify genes that have been upregulated, the blue ones indicate those that have gone down in expression, while the gray ones denote genes that haven’t changed. DEGs were defined as *p* < 0.05 and FC < 0.67 or FC > 1.5. (**B**) Top enriched signaling pathways of upregulated and downregulated DEGs. (**C**) Co-enriched pathways of differential genes and metabolites. (**D**) Relative levels of glutathione metabolism-related metabolites and genes. Relative levels were calculated by dividing the value of each sample by the mean value of the FC group. Data are presented as the mean ± SEM. Panels for L-glutathione (reduced) and Cys-Gly represent metabolite relative abundance, whereas panels for OPLAH, ANPEP, CHAC1, and GPX3 represent relative mRNA expression. Statistical analysis was performed using Student’s *t*-test. * *p* < 0.05, *** *p* < 0.001, **** *p* < 0.0001. (**E**) Schematic diagram of glutathione metabolism based on metabolomic and transcriptomic co-enriched pathways. Circles represent metabolites; rectangles represent genes. Green indicates downregulation. Red indicates upregulation. Arrows indicate the biochemical reaction direction.

To investigate changes in gene expression in CCs isolated from vitrified versus fresh porcine GV oocytes post-IVM, RNA-seq analysis was performed. In total, 741 DEGs were found when comparing the two groups, comprising 491 upregulated and 250 downregulated genes (Figure 5A). Next, KEGG pathway enrichment analysis was conducted. Upregulated genes were significantly enriched in 54 pathways, with major pathways including metabolic pathways, glutathione metabolism, and the cyclic adenosine monophosphate (cAMP) signaling pathway (Figure 5B, Supplemental Table S7). Downregulated genes demonstrated enrichment across 10 pathways, notably MAPK and Notch signaling pathways (Figure 5B, Supplemental Table S7).

To further characterize the effect of vitrification on CCs, integrated transcriptomic and metabolomic analyses were performed. Seven pathways were significantly enriched, including glutathione metabolism and glycerophospholipid metabolism (Figure 5C). In the glutathione metabolism pathway, GSH and Cys-Gly were significantly downregulated (Figure 5D,E).

In the glycerophospholipid metabolism pathway, all metabolites except phosphatidylcholine (PC), including LysoPC, LysoPE, lysophosphatidylserine (LysoPS), and lysophosphatidylinositol (LysoPI), were upregulated (Figure S6A,B).

### 3.6. Effects of Cys-Gly on the Development of Vitrified GV Oocytes

**Figure 6:**
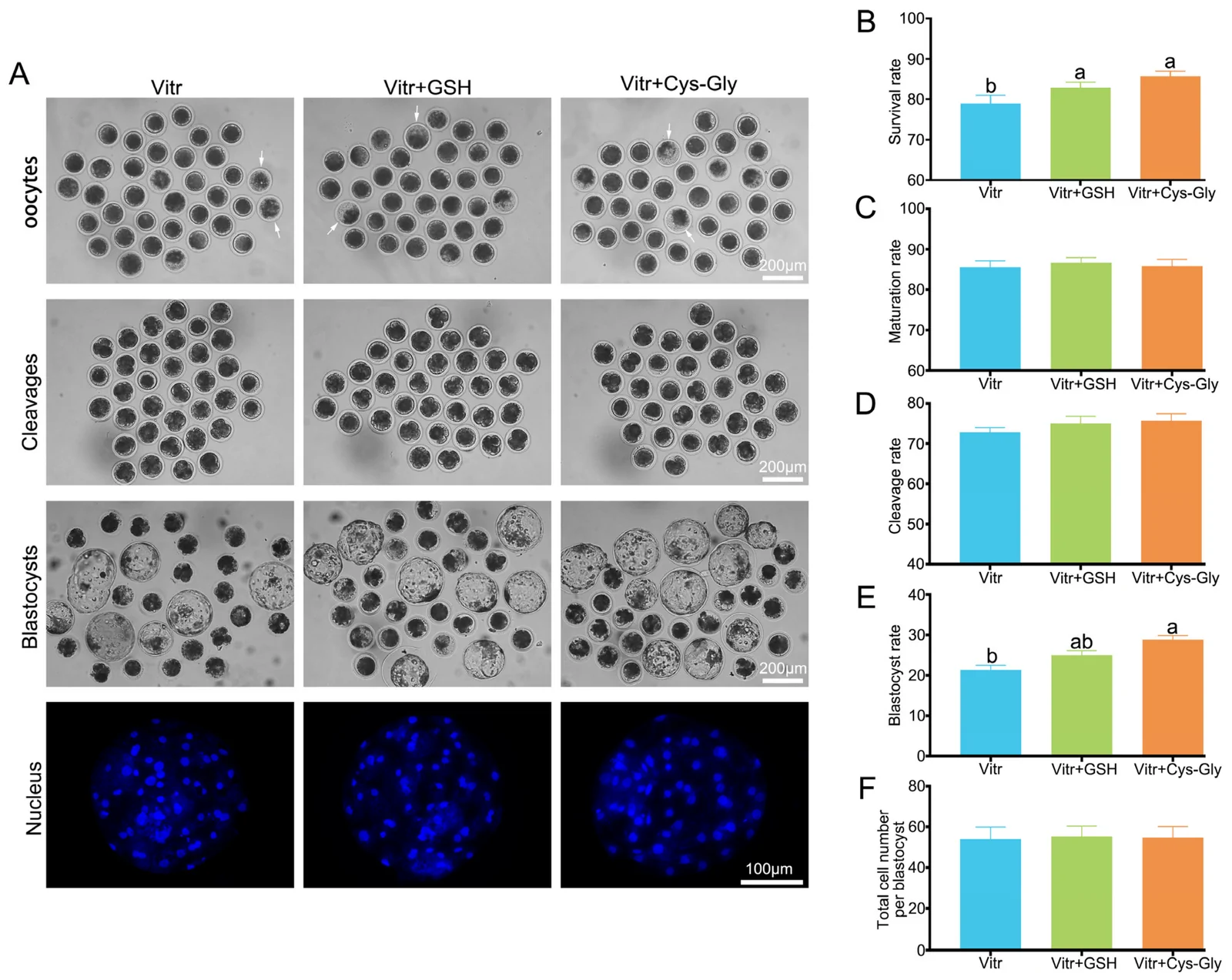
Effects of glutathione (GSH) or cysteinyl-glycine (Cys-Gly) on oocyte maturation and embryonic development of vitrified porcine GV oocytes. (**A**) Representative images of oocytes, cleavages, and blastocysts following parthenogenetic activation, stained with Hoechst33342. White arrows indicate non-viable oocytes. (**B**) Survival rate. (**C**) Nuclear maturation rate. (**D**) Cleavage rate. (**E**) Blastocyst formation rate. (**F**) Total cell number per blastocyst. Bars with different letters denote significant differences (*p* < 0.05). Results are presented as mean ± SEM.

Based on the integrated analyses, Cys-Gly and GSH were significantly downregulated in the oocytes and CCs after vitrification and IVM. Therefore, we investigated whether Cys-Gly supplementation could function as a potential antioxidant to improve the quality of vitrified oocytes, while GSH was included as a reference antioxidant control. As illustrated in Figure 6A,B, the survival rates of oocytes in Vitr + Cys-Gly (Vitrification + 2 mM Cys-Gly) and Vitr + GSH (Vitrification + 4 mM GSH) groups were notably better than those in the Vitr group. In contrast, no discernible differences in nuclear maturation rates were observed among the experimental groups (Figure 6C). After PA, cleavage rates did not differ significantly among the groups (Figure 6A,D). Nevertheless, the Vitr + Cys-Gly demonstrated a markedly enhanced blastocyst formation rate compared to the Vitr group (Figure 6A,E). When it came to the total cell count within blastocysts, no meaningful distinctions emerged across any of the experimental conditions (Figure 6A,F).

### 3.7. Effects of Cys-Gly on Oxidative Stress in Vitrified GV Oocytes

**Figure 7:**
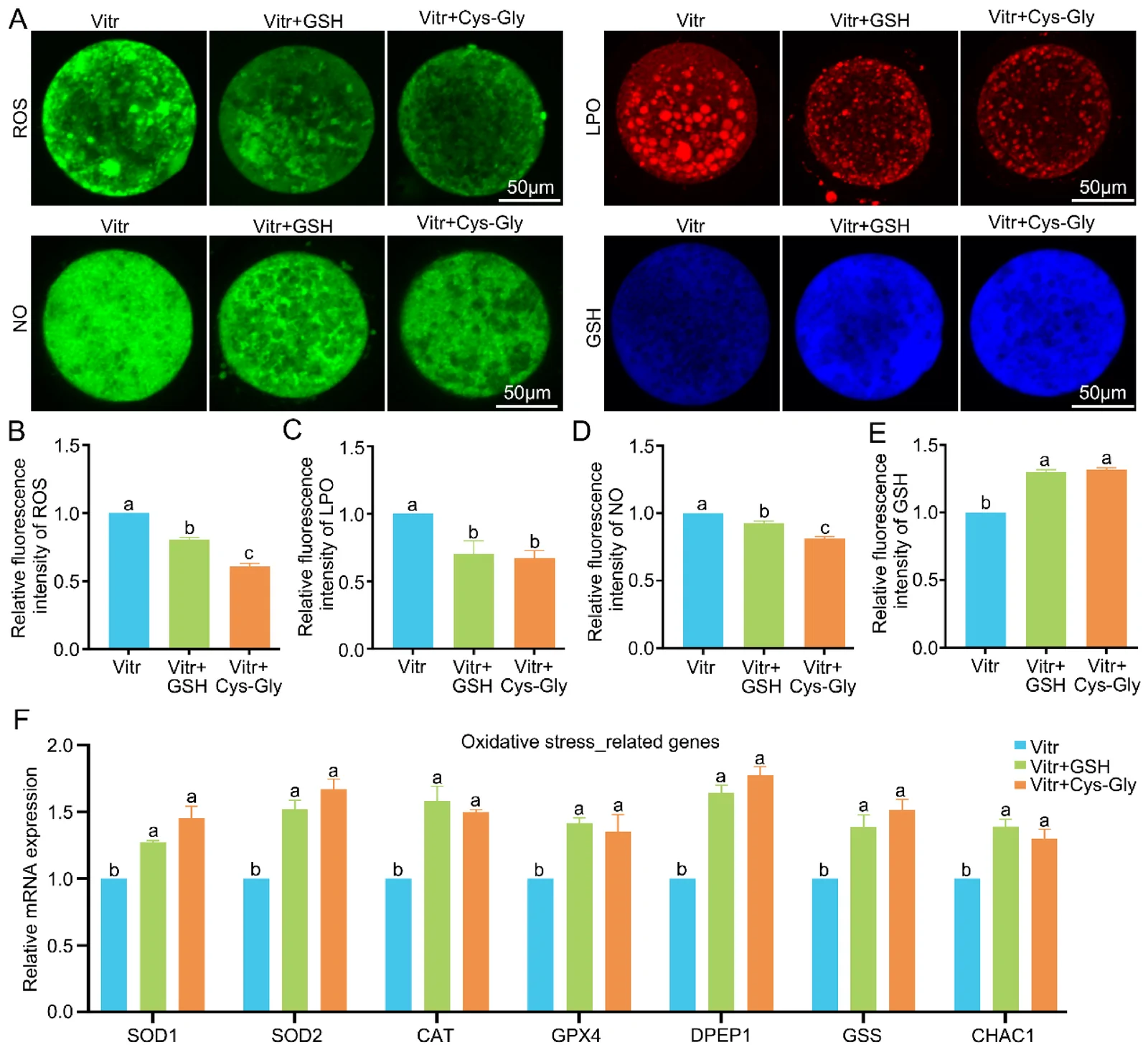
Effects of glutathione (GSH) or cysteinyl-glycine (Cys-Gly) on oxidative stress in vitrified porcine GV oocytes. (**A**) Representative images of reactive oxygen species (ROS), lipid peroxidation (LPO), nitric oxide (NO), and glutathione (GSH) in oocytes. (**B**) ROS levels by relative fluorescence intensity. (**C**) LPO levels by relative fluorescence intensity. (**D**) NO levels by relative fluorescence intensity. (**E**) GSH levels by relative fluorescence intensity. (**F**) Relative mRNA expression of oxidative stress-related genes. Bars with different letters denote significant differences (*p* < 0.05). Results are presented as mean ± SEM.

To evaluate the effectiveness of Cys-Gly in reducing oxidative stress, we measured the intracellular ROS, LPO, NO, and GSH levels at the MII stage of vitrified GV oocytes (Figure 7A). ROS, LPO, and NO levels were significantly reduced in Vitr + Cys-Gly and Vitr + GSH groups compared with the Vitr group, indicating that Cys-Gly and GSH exert antioxidant effects in vitrified oocytes (Figure 7A–D). The level of GSH was significantly increased in Vitr + Cys-Gly and Vitr + GSH groups compared with the Vitr group (Figure 7E). Finally, the relative mRNA abundance of genes tied to oxidative stress response and glutathione biosynthesis was evaluated using qRT-PCR. Compared with the Vitr group, Vitr + Cys-Gly and Vitr + GSH groups exhibited significantly increased expressions of these genes (Figure 7F and Figure S7).

### 3.8. Effects of Cys-Gly on Apoptosis and Autophagy in Vitrified GV Oocytes

**Figure 8:**
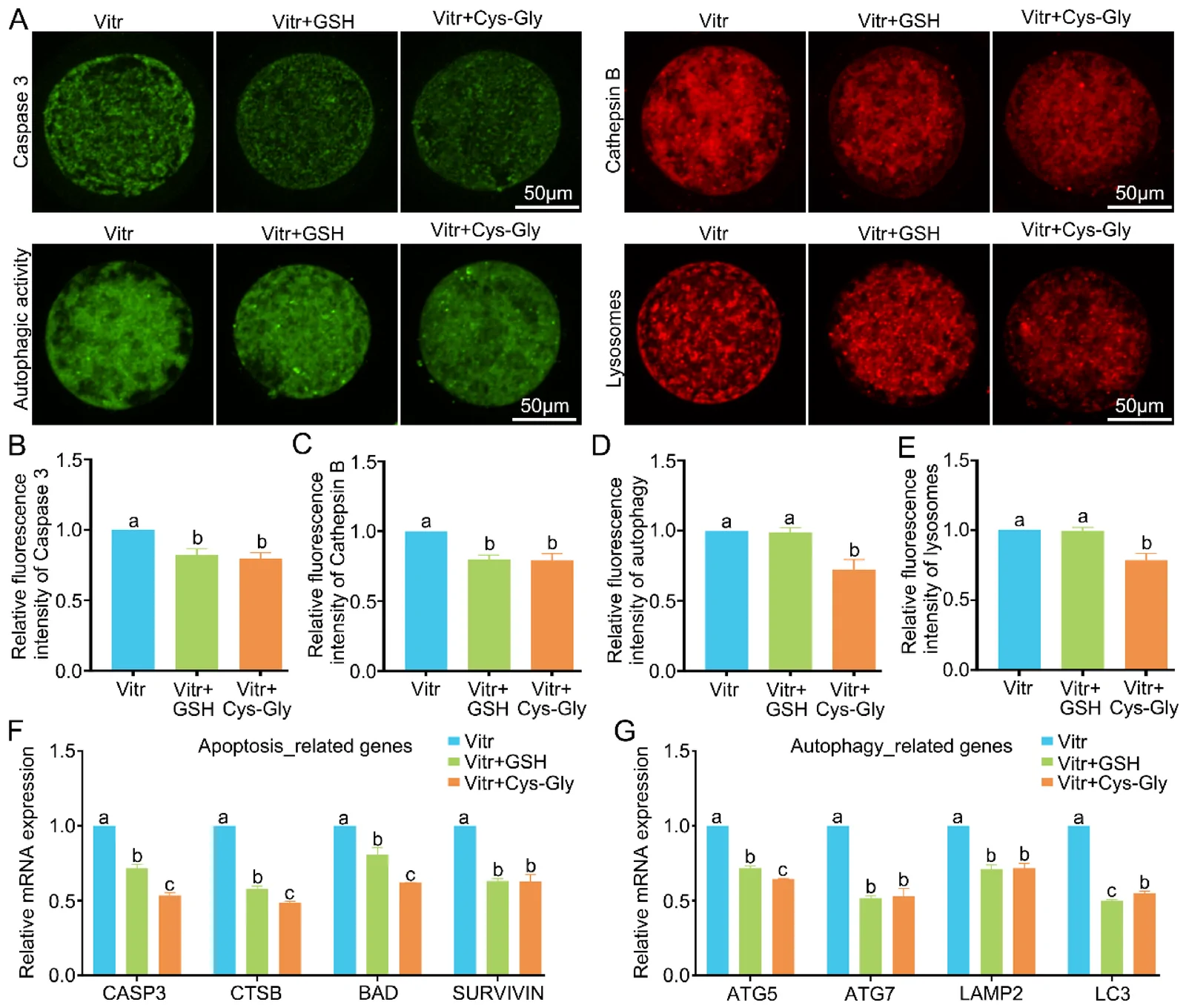
Effects of glutathione (GSH) or cysteinyl-glycine (Cys-Gly) on apoptosis and autophagy in vitrified porcine GV oocytes. (**A**) Representative images of caspase 3 activity, cathepsin B activity, autophagic activity, and lysosome activity in oocytes. (**B**) Caspase 3 activities by relative fluorescence intensity. (**C**) Cathepsin B activities by relative fluorescence intensity. (**D**) Autophagic activities by relative fluorescence intensity. (**E**) Lysosome activity by relative fluorescence intensity. (**F**) Relative mRNA expression of apoptosis-related genes. Bars with different letters denote significant differences (*p* < 0.05). Results are presented as mean ± SEM. (**G**) Relative mRNA expression of autophagy-related genes. Bars with different letters denote significant differences (*p* < 0.05). Results are presented as mean ± SEM.

To expore the influences of Cys-Gly on apoptosis and autophagy, we analyzed relevant apoptotic and autophagic levels in MII oocytes from vitrified GV oocytes (Figure 8A). Caspase3 and cathepsin B levels were significantly reduced in Vitr + Cys-Gly versus Vitr + GSH groups compared with the Vitr group (Figure 8A–C). In addition, autophagy and lysosomal activity were markedly decreased in Vitr + Cys-Gly groups relative to the Vitr group (Figure 8D–E). Furthermore, mRNA expression levels of apoptosis- and autophagy-related genes were also quantified. Compared with the Vitr group, Vitr + Cys-Gly and Vitr + GSH groups exhibited significantly decreased expressions of these genes (Figure 8F–G).

### 3.9. Effects of Cys-Gly on Cumulus Expansion

**Figure 9:**
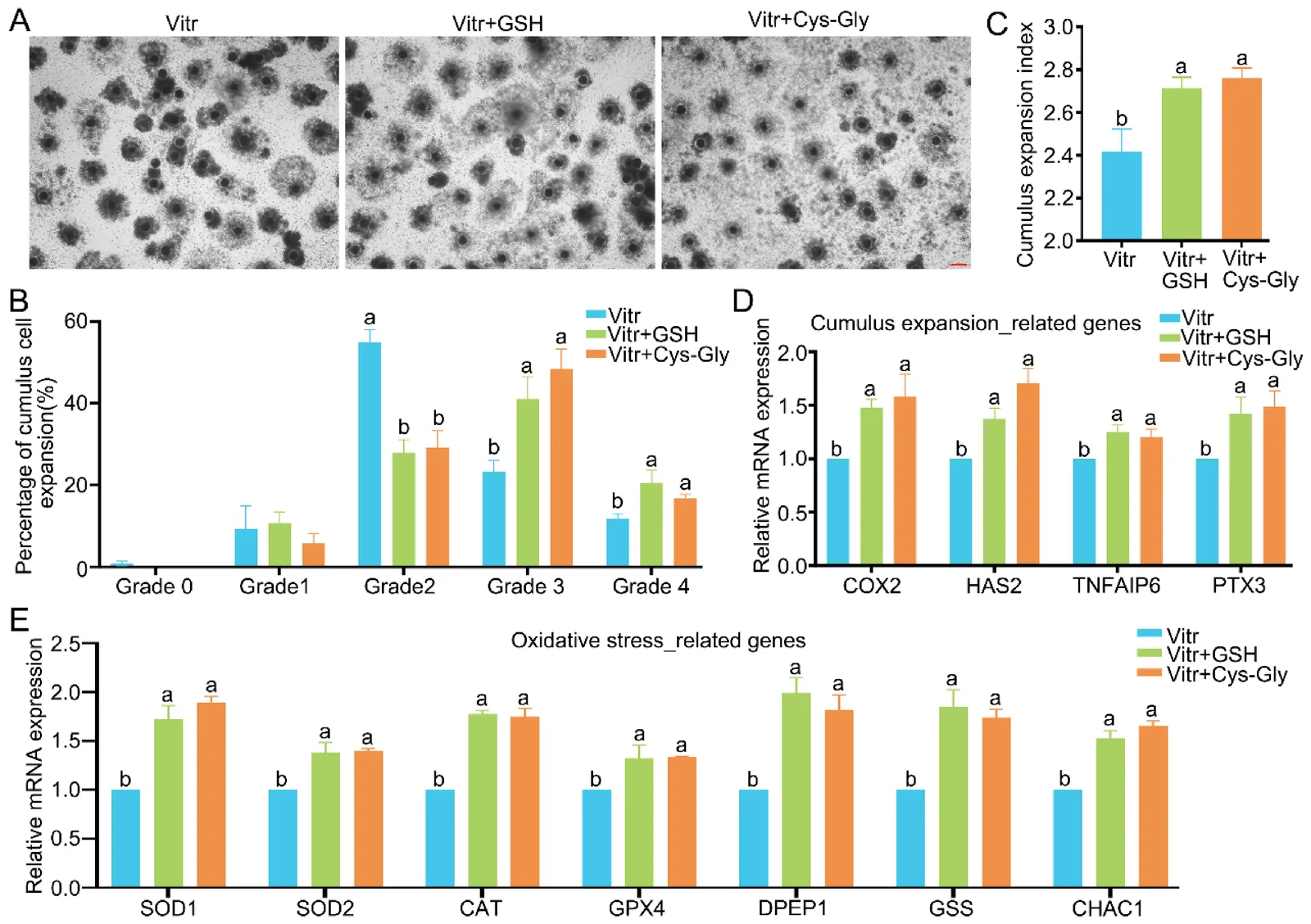
Effects of glutathione (GSH) or cysteinyl-glycine (Cys-Gly) on CC expansion. (**A**) Representative image of cumulus expansion after IVM of vitrified GV oocytes. Scale bar = 200 μm. (**B**) Different degrees of cumulus expansion (%). (**C**) Total cumulus expansion index. (**D**) Relative mRNA expression of cumulus expansion-related genes. (**E**) Relative mRNA expression of oxidative stress-related genes. Bars with different letters denote significant differences (*p* < 0.05). Results are presented as mean ± SEM.

We found that supplementation with GSH or Cys-Gly during IVM effectively improved CC expansion (Figure 9A). As depicted in Figure 9B, the vitrification (Vitr) + Cys-Gly and Vitr + GSH groups significantly increased the proportions of COCs with CEI grades 3 and 4 relative to the Vitr group, whereas the proportion of COCs with CEI grade 2 was significantly decreased. Consistently, overall CEI was significantly higher in Vitr + Cys-Gly and Vitr + GSH groups than in the Vitr group (Figure 9C). To further validate the effects of Cys-Gly and GSH on CC expansion, changes in the expression levels of cumulus expansion genes were evaluated. Vitr + Cys-Gly and Vitr + GSH groups exhibited significant upregulation of these genes versus the Vitr group (Figure 9D). In addition, oxidative stress- and glutathione metabolism-related genes were significantly upregulated in Vitr + Cys-Gly and Vitr + GSH groups versus the Vitr group (Figure 9E).

## 4. Discussion

Given their elevated lipid stores and inherent fragility when exposed to cold, por-cine oocytes are particularly prone to injury during vitrification, including osmotic shock and oxidative damage. These stressors can severely undermine their develop-mental potential or even trigger outright cell death. Currently, research on oocyte cryodamage mainly focuses on genomic and proteomic alterations, while metabolic-level investigations are limited. In this study, UPLC-MS-based metabolomic and transcriptomic profiling were integrated to comprehensively assess the effects of vitrification on porcine GV-stage oocytes and CCs after IVM. Our multi-omics analysis revealed extensive alterations in metabolic profiles and gene expression alongside reduced endogenous Cys-Gly in vitrified oocytes and CCs. Exogenous Cys-Gly supplementation alleviated oxidative damage and improved CC expansion and embryonic development. These findings provide mechanistic elucidate cryopreservation-induced stress and its implications for oocyte developmental competence.

Oocyte maturation is essential for obtaining high-quality embryos, and precise regulation of metabolic processes plays an important role in this process [32,33]. In our study, metabolomic–transcriptomic analysis revealed that vitrified oocytes underwent extensive metabolic reprogramming compared with fresh oocytes, primarily characterized by disruptions in energy metabolism, oxidative stress regulation, and phospholipid metabolism. Oocyte maturation involves both nuclear and cytoplasmic maturation [34], and asynchrony between these processes can compromise oocyte quality and impair subsequent embryo development [35,36,37]. Oocyte maturation is an energy-dependent process, with glycometabolism serving as its primary energy source. The pentose phosphate pathway plays a particularly important role in promoting oocyte cytoplasmic maturation by reducing oxidative stress and providing D-fructose-6-phosphate (F6P) for glycolysis [38]. Inhibition of the pentose phosphate pathway in porcine oocytes alters oxidative and mitochondrial dynamics, resulting in meiotic arrest [39]. Glucose metabolism intermediates also regulate intracellular signaling and osmotic balance to ensure the maturation of the oocyte nucleus and cytoplasm. Our study revealed abnormal expression of multiple carbohydrate metabolism intermediates. In particular, vitrification resulted in reduced expression of key pentose phosphate pathway intermediates, including F6P, xylulose 5-phosphate, and ribulose-5-phosphate. A surge in cAMP in the follicle and its subsequent conversion to AMP promotes oocyte maturation and ovulation [40]. Our data indicated that vitrified GV oocytes exhibited altered levels of AMP, accompanied by downregulation of CREB5 expression. GSH plays an vital role in maintaining the morphology of the meiotic spindle and supporting embryo development [41,42]. GSS and GSH were significantly downregulated in vitrified oocytes, which may affect subsequent embryo development. Alterations in lipid metabolism also influence oocyte development [43]. We observed reduced expression of phospholipid-related metabolites, including LPC and LPE. Previous research indicates that LPC plays an essential role in follicular development and oocyte maturation [44].

The effects of vitrification on oocyte energy metabolism are also reflected in metabolic reprogramming within CCs. In this study, several metabolites associated with energy metabolism were significantly upregulated in vitrified CCs, including AMP, F6P, and phospholipids involved in membrane synthesis. This shift indicates that CCs may compensate for insufficient energy supply to oocytes during vitrification by enhancing the synthesis and transport of energy-related metabolites, thereby preserving the functional integrity of COCs. Furthermore, we observed activation of the PI3K-Akt signaling pathway in vitrified CCs. As a highly conserved regulatory pathway, PI3K-Akt inhibits pro-apoptotic protein activity and promotes anti-apoptotic protein expression, significantly enhancing oocyte anti-apoptotic capacity. Akt activation promotes glucose transporter expression, thereby enhancing CC glucose uptake and glycolytic efficiency and providing the energy required for oocyte meiosis [45]. Accordingly, activation of the PI3K-AKT signaling pathway in vitrified CCs reduces apoptosis caused by cryoinjury while enhancing glycolytic efficiency, thereby ensuring sufficient energy supply for post-warming oocyte maturation. To further characterize metabolic regulation in vitrified CCs, integrated metabolomic and transcriptomic analyses were performed. Two key pathways were jointly enriched: glutathione metabolism and glycerophospholipid metabolism. Changes in GSH-related metabolites in CCs were consistent with those observed in oocytes, suggesting a synergistic regulatory mechanism between CCs and oocytes in antioxidant stress response. This synchronized regulation may contribute to the joint clearance of ROS generated during vitrification, thereby mitigating the impact of oxidative damage on oocyte development. Conversely, glycerophospholipid metabolites in CCs exhibited patterns opposite to those observed in oocytes. This implies that CCs may maintain membrane stability through remodeling of glycerophospholipid synthesis and metabolism, thereby contributing to structural protection of the oocyte.

KEGG enrichment analysis further revealed significant enrichment of ferroptosis, GSH, and AMPK signaling pathways among downregulated metabolites in vitrified oocytes and CCs. Recent studies have found that ferroptosis inhibition enhances fertility in aged females and that the main cause of ferroptosis in aged oocytes is a significant decrease in FTH1 expression [46]. Our data demonstrated that FTH1 was significantly downregulated in vitrified oocytes, which may contribute to reduced oocyte quality following vitrification. Integrated metabolomic analysis revealed that vitrification also resulted in significant dysregulation of GSH metabolism in oocytes and CCs. In CCs, the expression of GSH-associated genes, including GSTM3, GPX3, and MGST1, was markedly increased. This may be because vitrification triggers intracellular oxidative stress, and GSH is involved in the antioxidant response that protects the cells from oxidative stress damage [47]. GSH is a cofactor for GPX4, a lipid hydroperoxidase that inhibits ferroptosis [48]. No dysregulation of GPX4 expression was observed in vitrified oocytes and CCs. Additional studies have identified AIFM2, also known as FSP1, as a GSH-independent inhibitor of ferroptosis [49]. In our dataset, AIFM2 was downregulated 2.7-fold in vitrified oocytes compared with fresh oocytes. AMPK pathway serves as a metabolic balance regulator, initiating during physiological or pathological stresses to restore energetic equilibrium [50]. Enrichment of downregulated metabolites in the AMPK signaling pathway in both vitrified oocytes and CCs indicates a disruption in energy balance.

Cys-Gly is a key dipeptide intermediate in the metabolic cycle of GSH. Subsequently, it is hydrolyzed by Cys-Gly dipeptidase into cysteine and glycine, providing the rate-limiting substrate for the resynthesis of GSH [51]. Cys-Gly occupies a pivotal position in GSH metabolism, but there are currently very limited studies directly focusing on the relationship between Cys-Gly and oocytes or CCs. Our research results show that the supplementation of both Cys-Gly and GSH can improve the quality of vitrified porcine oocytes. These beneficial effects were reflected in two key aspects. First, both treatments enhanced CC expansion. Previous studies indicates that CC expansion is a pivotal marker of oocyte maturation and is closely related to oocyte quality and developmental potential [52]. Second, Cys-Gly supplementation effectively enhanced oocyte quality and supported embryonic development. We found that oocyte survival was significantly improved by supplementation with Cys-Gly or GSH during IVM, indicating their protective effect on vitrified oocytes. This is consistent with previous reports showing that supplementation with GSH or its precursors during oocyte vitrification or IVM improves oocyte developmental competence [47,53,54,55]. Supplementation with Cys-Gly or GSH significantly reduced ROS, LPO, and NO levels while markedly increasing intracellular GSH content. GSH supplementation can ensure normal nuclear and cytoplasmic maturation of oocytes by suppressing ROS production and apoptosis [30]. Consistently, our results suggest that Cys-Gly and GSH may directly scavenge intracellular free radicals, reduce oxidative stress damage, and increase intracellular antioxidant levels, thereby enhancing the antioxidant defense capacity of vitrified oocytes. In addition, supplementation with Cys-Gly or GSH enhanced oxidative stress gene expression in both oocytes and cumulus cells, which may reflect an adaptive response of the endogenous antioxidant defense system. Furthermore, we also found that the addition of Cys-Gly can significantly reduce apoptosis and autophagy, which further indicates that it helps maintain the redox balance within the cells and mitigating oxidative stress-induced cellular damage. The above research evaluated the potential protective effect of Cys-Gly on vitrified porcine oocytes. The results revealed that, apart from directly supplementing the end-product (GSH), regulating its key metabolic intermediate (Cys-Gly) may be a more effective strategy for improving oocyte quality after vitrification.

## 5. Conclusions

In conclusion, 63 metabolites in oocytes and 91 metabolites in CCs were significantly differentially expressed following vitrification and subsequent IVM of porcine GV oocytes. Metabolic expression variations may reveal insights into the diminished developmental potential of cryopreserved oocytes. Integrated metabolomic and transcriptomic analyses revealed that vitrification disrupts energy metabolism, oxidative stress regulation, and phospholipid metabolism, which may collectively impair embryonic development. Supplementation with exogenous Cys-Gly demonstrated beneficial effects by enhancing CC expansion and improving oocyte quality and developmental potential, providing a potential strategy for optimizing vitrification. These findings provide mechanistic insights into the metabolic basis of reduced developmental competence in vitrified GV oocytes and provide a basis for improving oocyte quality and refining in vitro culture protocols.

## Data Availability

The authors declare that the data supporting the findings of this study are available within the paper. RNA-seq data have been uploaded to NCBI (BioProject: PRJNA1538753). Metabolomics data have been deposited in the National Genomics Data Center (NGDC) OMIX database under accession number OMIX021511.

## Supplementary Materials

The following supporting information can be downloaded at: https://www.mdpi.com/article/10.3390/antiox15101294/s1, Figure S1: Identification of metabolites of oocytes and cumulus cells; Figure S2: Differential metabolite enrichment pathways shared by VO vs FO and VC vs FC; Figure S3: Differential metabolite classification ring diagram based on matching results against the HMDB database; Figure S4: Energy metabolism alterations in vitrified and fresh porcine GV oocytes post-IVM; Figure S5: Glycerophospholipid metabolism in vitrified and fresh porcine GV oocytes post-IVM; Figure S6: Glycerophospholipid metabolism in CCs from vitrified and fresh porcine GV oocytes post-IVM; Figure S7: The cycle threshold (Ct) of the reference gene in different groups; Table S1: qPCR primers, survival rates, nuclear maturation and embryonic development; Table S2: VO vs FO_diff_MS2_metabolites; Table S3: VO vs FO DEMs enrichment result; Table S4: VO vs FO gene enrichment result; Table S5: VC vs FC_diff_MS2_metabolites; Table S6: VC vs FC DEMs enrichment result; Table S7: VC vs FC gene enrichment result.

## Abbreviations

The following abbreviations are used in this manuscript:

GV	Germinal Vesicle
CCs	Cumulus Cells
IVM	In Vitro Maturation
Cys-Gly	Cysteinyl-glycine
COCs	Cumulus–oocyte complexes
ROS	Reactive oxygen species
LPO	Lipid peroxidation
NO	Nitric oxide
GSH	Glutathione
