# Supplementary material for: Metabolomic and Transcriptomic Changes in Porcine Cumulus–Oocyte Complexes Following Vitrification: Cysteinyl-Glycine as an Antioxidant Enhances the Quality of Vitrified Oocytes
Source: Antioxidants (Basel). 2026 Oct 5;15(10):1294. doi: 10.3390/antiox15101294 (PMC13701736; doi:10.3390/antiox15101294)
Supplement: Supplementary file 1 [file antioxidants-15-01294-s001.zip › Supplementary Figures.pdf]

Supplementary Data

Metabolomic and Transcriptomic Changes in Porcine Cumulus–Oocyte Complexes Following Vitrification: Cysteinyl-Glycine as an Antioxidant Enhances the Quality of Vitrified Oocytes

Zhen He [1,2,†], Decai Xiang [1,†], Jing Fu [1], Demeng Mu [1], Xuemei Li [2], Junyu Jiang [2], Hongjiang Wei [2], Baoyu Jia [2,*] and Guoquan Wu [1,*]

[1] National Regional Genebank (Yunnan) of Livestock and Poultry Genetic Resources, Yunnan Provincial
Engineering Laboratory of Animal Genetic Resource Conservation and Germplasm Enhancement, Yunnan Animal Science and Veterinary Institute, Kunming 650224, China; hezhen2026@163.com (Z.H.); askalm@163.com (D.X.); fj11260027@163.com (J.F.); mudemeng1219@163.com (D.M.)

[2] Key Laboratory for Porcine Gene Editing and Xenotransplantation in Yunnan Province, College of
Veterinary Medicine, Yunnan Agricultural University, Kunming 650201, China;
lxm19187386050@163.com (X.L.); 13987988411@163.com (J.J.); hongjiangwei@126.com (H.W.)

* Correspondence: jiabaoyu2009@163.com (B.J.); wuguoquan1982@163.com (G.W.)

† These authors contributed equally to this work.

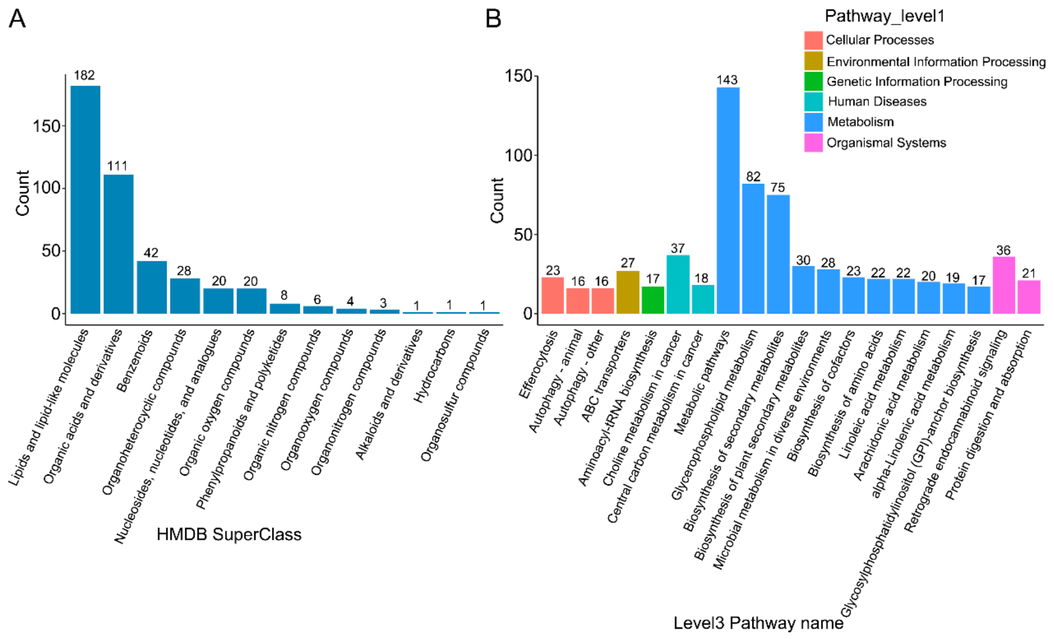

Figure S1. Identification of metabolites of oocytes and cumulus cells. (A) HMDB Super class classification diagram. Super class entries are used as horizontal coordinates and the number of metabolites corresponding to the entries as vertical coordinates. (B)KEGG pathway classification diagram. KEGG pathway secondary entries are shown as horizontal coordinates, and the number of metabolites corresponding to the entries are shown as vertical coordinates, and KEGG pathway primary entries are shown separately and differentiated by different colors.

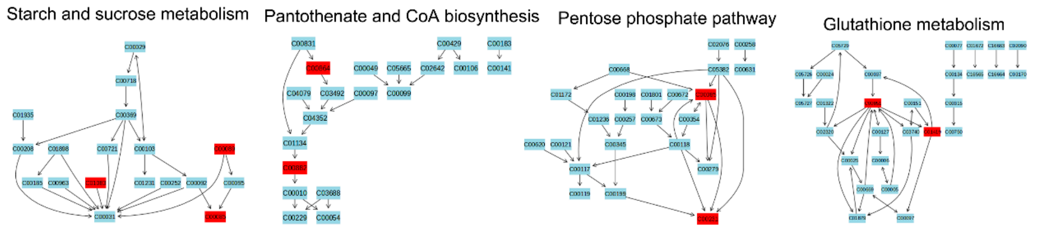

FigureS2. Differential metabolite enrichment pathways shared by VO vs FO and VC vs FC.

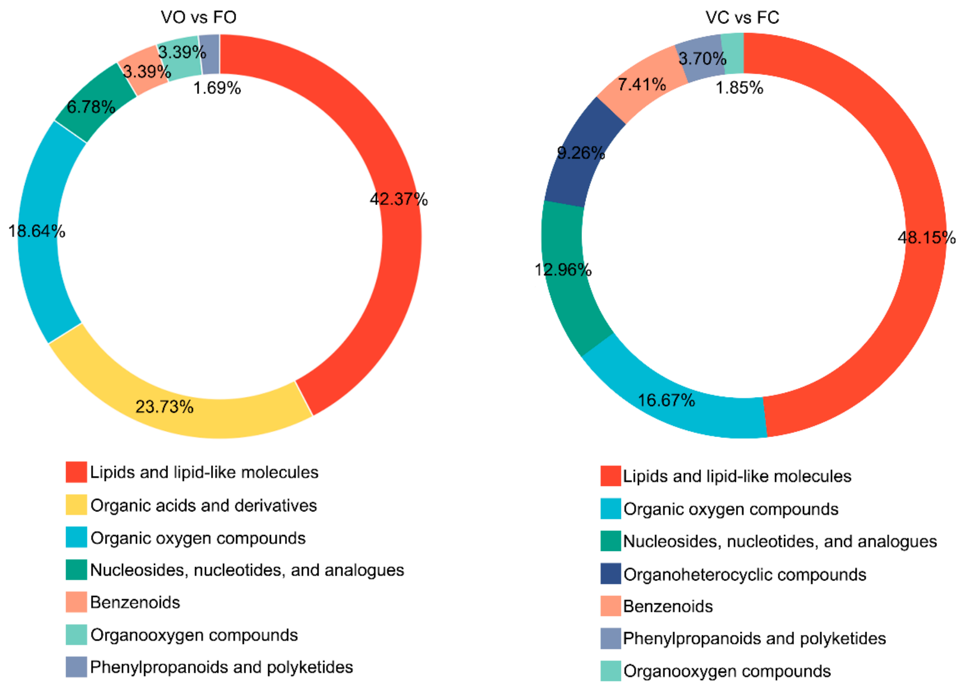

Figure S3. Differential metabolite classification ring diagram based on matching results against the HMDB database.

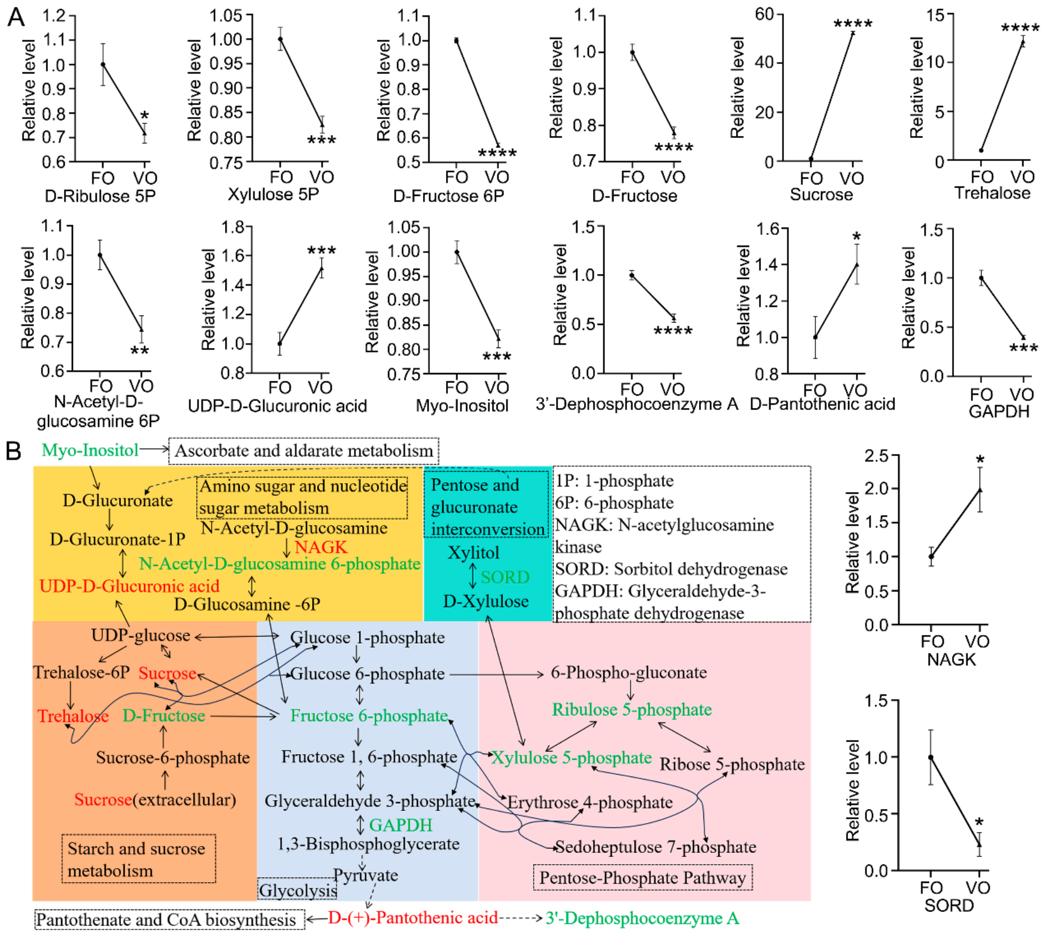

Figure S4. Energy metabolism alterations in vitrified and fresh porcine GV oocytes post-IVM. (A) Relative levels of metabolites and genes involved in energy metabolism. Error bars indicate SEM. Statistical analysis was performed using Student's t-test. (B) Schematic diagram of energy metabolism from integrated metabolomics and transcriptomics analysis. Green indicates downregulated metabolites or genes; red indicates upregulated metabolites or genes.

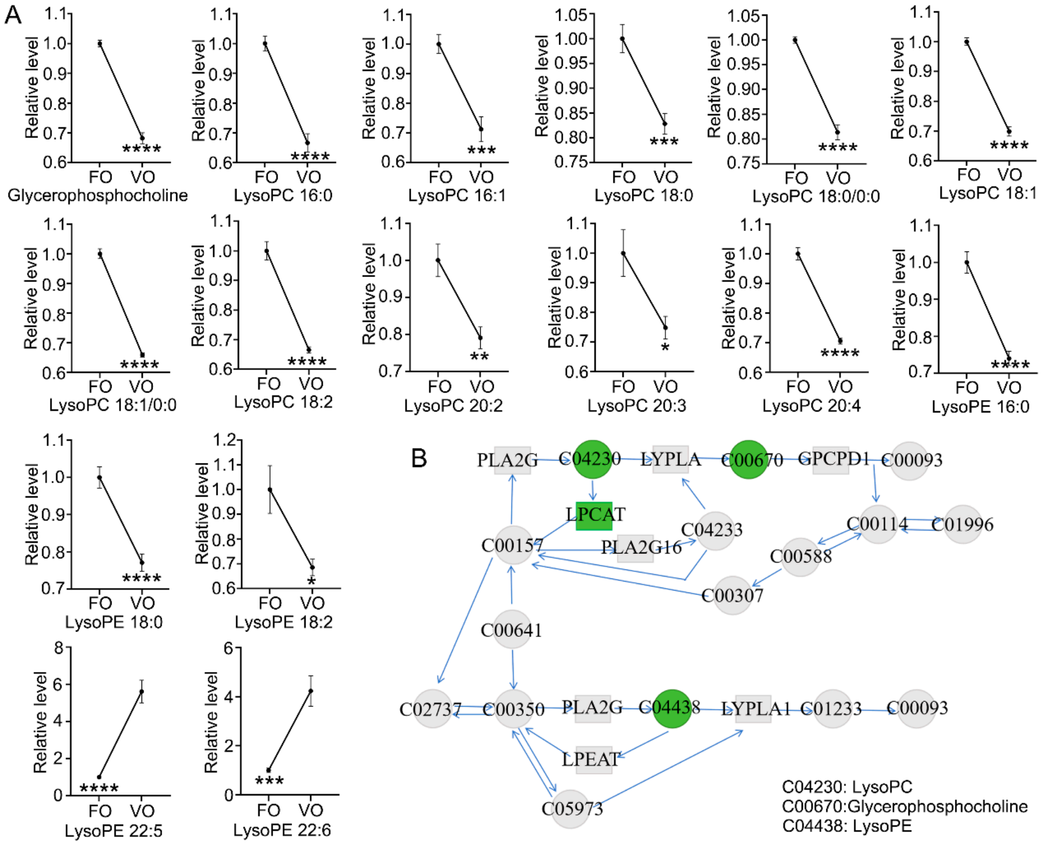

Figure S5. Glycerophospholipid metabolism in vitrified and fresh porcine GV oocytes post-IVM. (A) Relative levels of metabolites and genes involved in glycerophospholipid metabolism. Error bars indicate SEM. Statistical analysis was performed using Student's t-test. (B) Schematic diagram of glycerophospholipid metabolism based on metabolomics and transcriptomics co-enriched pathways. Circles represent metabolites; rectangles represent genes. Green indicates downregulated metabolites or genes.

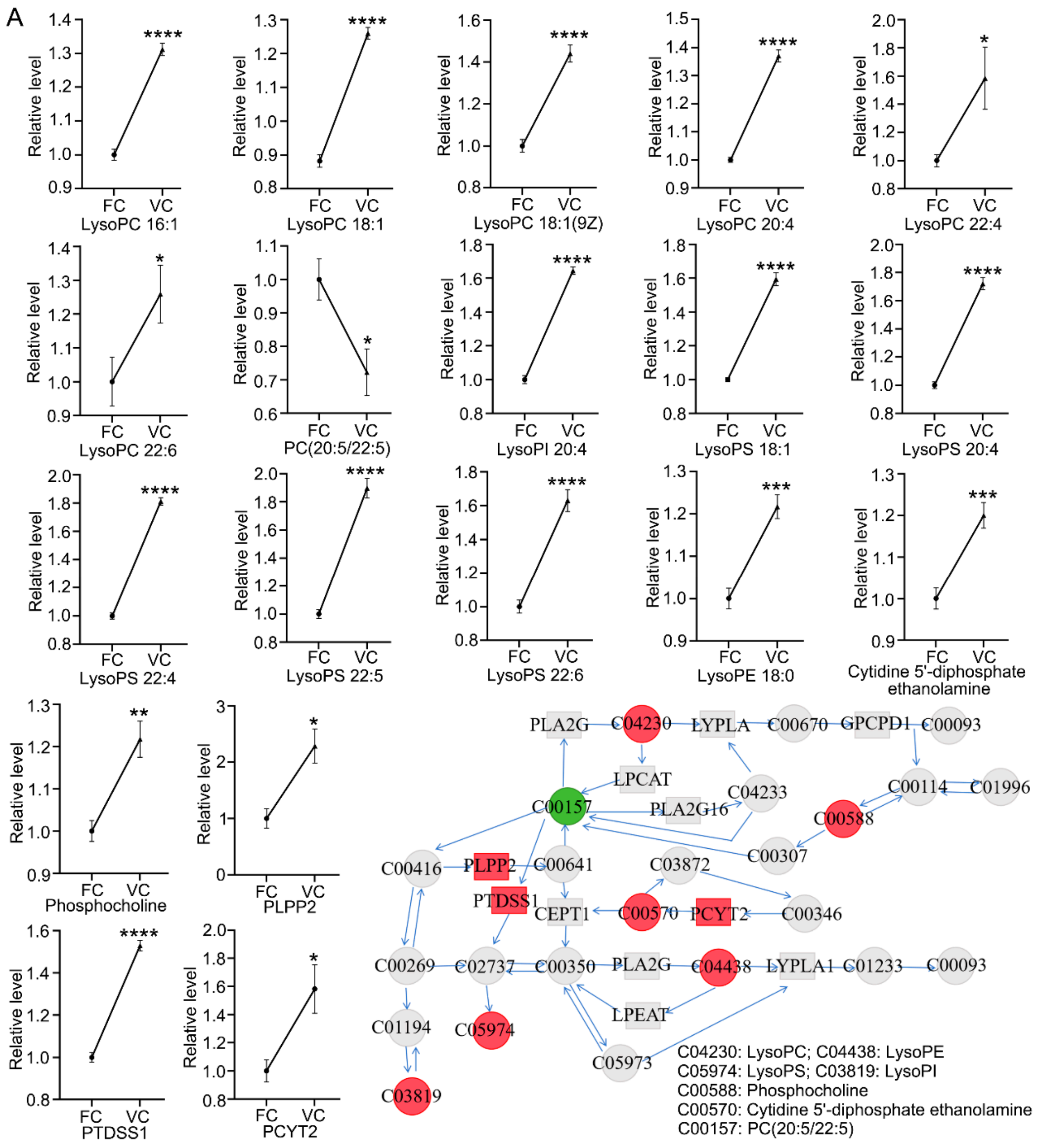

Figure S6. Glycerophospholipid metabolism in CCs from vitrified and fresh porcine GV oocytes post-IVM. (A) Relative levels of glycerophospholipid metabolism-related metabolites and genes. Error bars indicate SEM. Statistical analysis was performed using Student's t-test. (B) Schematic diagram of glycerophospholipid metabolism based on metabolomics and transcriptomics co-enriched pathways. Circles represent metabolites; rectangles represent genes. Green indicates downregulated metabolites or genes; red indicates upregulated metabolites or genes.

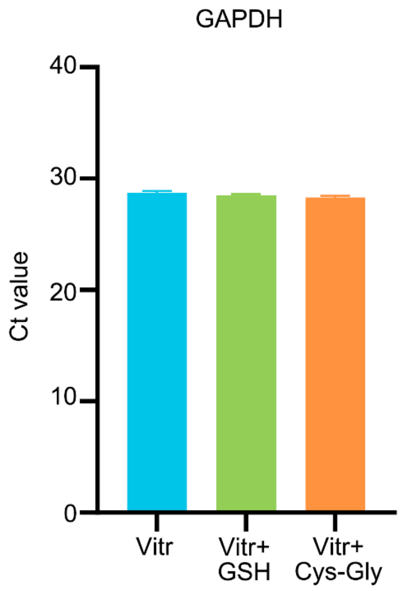

Figure S7. The cycle threshold (Ct) of the reference gene in different groups. Ct values were obtained by quantitative real-time PCR to verify the stability of the internal reference gene. Bars represent mean ± SEM. Statistical analysis showed no significant difference between groups.
