# Supplementary material for: Metabolomic and Transcriptomic Changes in Porcine Cumulus–Oocyte Complexes Following Vitrification: Cysteinyl-Glycine as an Antioxidant Enhances the Quality of Vitrified Oocytes
Source: Antioxidants (Basel). 2026 Oct 5;15(10):1294. doi: 10.3390/antiox15101294 (PMC13701736; doi:10.3390/antiox15101294)
Supplement: Supplementary file 1 [file antioxidants-15-01294-s001.zip › Supplementary Table S1 qPCR primer, survival rates, nuclear maturation and embryonic development.pdf]

Primers(5’-3’) used in this study

mRNA		Primer sequence(5’-3’)
COX2	F	GGCTGCGGGAACATAATAGA
	R	GCAGCTCTGGGTCAAACTTC
HAS2	F	TTACAATCCTCCTGGGTGGT
	R	TCAAGCACCATGTCGTACTG
TNFAIP6	F	TACGACAGTTACGACGACGT
	R	CACGGAAGCATCACTTAGGA
PTX3	F	AGACTTTATGCCATGGTGCT
	R	TGACAGTGAGCAATGAACAA
SOD1	F	TCCATGTCCATCAGTTTGGA
	R	AGTCACATTGCCCAGGTCTC
SOD2	F	AAGCCATCAAACGCGACTTT
	R	CCTTGTTGAAACCGAGCCAA
CAT	F	ACATGGTCTGGGACTTCTGG
	R	TCATGTGCCTGTGTCCATCT
GPX4	F	ATTCTCAGCCAAGGACATCG
	R	CCTCATTGAGAGGCCACATT
DPEP1	F	GGACGTGTCCAAGTACCCAG
	R	CCTGGAACCTGAGCGTGATT
GSS	F	AGTGTTGCTAAGGACCTCGC
	R	GAGGGGAAGAGTGTGAACGG
CHAC1	F	AGTGACAAGATGCCTGGTCG
	R	TCATTCACCTGCTCTCCACG
CASPASE3	F	GAGGCAGACTTCTTGTATGC
	R	CATGGACACAATACATGGAA
CTSB	F	CTCTAGGAACGAGAAGGAGAT
	R	CCAGACTTATACTGCAGGAAG
BAD	F	GAGGCAGACTTCTTGTATGC
	R	CATGGACACAATACATGGAA
SURVIVIN	F	CTGCAAAGAAAGTCCGCTGT
	R	TTCAAAAGGCTGGCGTTCTC
ATG5	F	ACCACTGCTTTCCTCCACTG
	R	CCATGGTCACCTCAGGAAAT
ATG7	F	GAACGGGAAGGATTTAATTT
	R	CAGTCAAGTCCTCCAAGAAG
LAMP2	F	CACCCACTCCAAAGGAAAAA
	R	GGTTGTCGTTTTTCACAGCA
LC3	F	CCGAACCTTCGAACAGAGAG
	R	AGGCTTGGTTAGCATTGAGC
ATCB	F	TCAACACCCCAGCCATGTAC
	R	CTCCGGAGTCCATCACGATG
GAPDH	F	GACCCCTTCATTGACCTCCA
	R	TGGAAGATGGTGATGGCCTT

Overview of survival and nuclear maturation of fresh and vitrified porcine immature oocytes.

Groups	No. of oocytes	Survival rate after warming (%)	Survival rate after IVM (%)	Nuclear maturation rate (%)
Fresh	300	100.0 ± 0[a]	89.3 ± 1.7[a]	88.8 ± 1.5
Vitrified	450	85.6 ± 1.2[b]	80.5 ± 1.3[b]	85.5 ± 1.6

Data are expressed as mean ± SEM values.

[a,b]Different superscripts in the same column indicate significant differences ($P < 0.05$).

Survival rate = viable oocytes/total oocytes cultured.

Nuclear maturation rate = MII oocytes/surviving oocytes.

Overview of developmental competence of fresh and vitrified porcine immature oocytes after parthenogenetic activation.

Groups	No. of oocytes	Cleavage rate (%)	Blastocyst rate (%)
Fresh	160	88.1 ± 1.8[a]	54.4 ± 1.9[a]
Vitrified	190	73.2 ± 1.6[b]	22.1 ± 1.6[b]

Data are expressed as mean ± SEM values.

[a,b]Different superscripts in the same column indicate significant differences ($P < 0.05$).
